# A recurrent cancer-associated fibroblast/TGF-beta/endothelial barrier direction is associated with immune exclusion and checkpoint resistance in bladder cancer: a public multi-cohort transcriptomic study

**DOI:** 10.1371/journal.pone.0358043

**Published:** 2026-09-22

**Authors:** Yuye Wu, Guangjie Li, Lixing Jiang, Chaohong Zhang, Huilong Fang, Haiqing Fan

**Affiliations:** Department of Urology, The Second Affiliated Hospital of Fujian University of Traditional Chinese Medicine, Fuzhou, China; Mayo Clinic Cancer Center, UNITED STATES OF AMERICA

## Abstract

Immune checkpoint inhibitors benefit only a subset of patients with urothelial carcinoma, and bulk biomarkers may not capture spatially organized stromal and vascular programs that restrict immune access. We performed a retrospective public multi-cohort transcriptomic study to examine whether a cancer-associated fibroblast (CAF)/TGF-beta/endothelial barrier direction is associated with immune exclusion and checkpoint resistance in bladder cancer. GSE171351 was used for spatial discovery, GSE319536 for external spatial recurrence testing, TCGA-BLCA for prognosis-oriented support, and IMvigor210, GSE176307 and GSE328930/DUTRENEO for treatment-response projection. In the four-section discovery cohort, rank-5 non-negative matrix factorization separated an E2 barrier-enriched program from an E4 hypoxic epithelial program, although component assignment was strongly associated with section identity. The external cohort reproduced the direct CAF/TGF-beta/endothelial barrier direction in 22 of 22 sections but did not reproduce E2/E4 mutual exclusivity: projected E2 and E4 were positively correlated (rho = 0.928). In IMvigor210, top-quartile E2-high tumors showed suggestive, but not statistically conclusive, evidence of anti-PD-L1 non-response after full biologic covariate adjustment (OR=2.57, 95% CI 1.00–6.64, p = 0.051; delta AUC = 0.009). A prespecified heuristic barrier-exclusion score was associated with non-response in exploratory fixed- and random-effects summaries (OR=1.52, 95% CI 1.20–1.92), including a sensitivity analysis excluding GSE328930. These results identify a recurrent barrier direction rather than a universal two-niche architecture and support a hypothesis-generating mechanism-to-translation framework that requires prospective spatial and pathology-level validation.

## Introduction

Bladder cancer remains clinically heterogeneous, and muscle-invasive bladder cancer (MIBC) accounts for much of the relapse, metastatic progression and cancer-specific mortality associated with the disease [1,2]. Platinum-based therapy, radical local treatment and immune checkpoint inhibitor (ICI) therapy have changed the management of advanced and high-risk disease, yet durable benefit from PD-1/PD-L1 blockade remains restricted to a minority of patients [2–5]. A practical challenge is therefore to identify resistance programs that are biologically interpretable, measurable across cohorts and not reducible to a single bulk immune marker.

Current urothelial carcinoma biomarkers include tumor mutational burden (TMB), PD-L1 expression, molecular subtype and bulk immune-infiltration signatures [5–7]. These variables are informative, but they compress epithelial, stromal, endothelial and immune compartments into a sample-level average. Spatial and single-cell studies now show that bladder cancer contains compartmentalized epithelial and microenvironmental states, including spatially restricted tumor subpopulations, intratumor subtype heterogeneity, spatially variable MIBC microenvironments and distinct cancer-associated fibroblast (CAF) programs [8–13]. This raises the possibility that checkpoint resistance reflects tissue architecture as well as immune-cell abundance.

A stromal-barrier model is supported by several strands of evidence. In urothelial carcinoma, TGF-beta signaling has been linked to T-cell exclusion and attenuated response to PD-L1 blockade [6]. In broader cancer contexts, TGF-beta-mediated immune evasion, CAF-rich stromal programs, endothelial remodeling and tumor-associated vasculature can regulate immune entry, homing and retention [14–16]. Myeloid and macrophage/CD8 states also shape urothelial cancer progression and ICI resistance [17,18]. Recent spatial-ecotype approaches provide a way to model multicellular tissue organization rather than treating its components as isolated markers [19]. Spatial EcoTyper learns cell-type-specific expression covariation across large multisample collections before factorizing recurrent ecotypes, whereas the present analysis applies locked biologic modules and local-neighborhood summaries within four GSE171351 sections. The approaches are related in concept but methodologically distinct. The recent GSE319536 MIBC atlas describes lineage-associated tumor states and immune architecture across a larger spatial cohort [20]; here, that resource is used to test a narrower, predefined barrier direction rather than to rediscover the atlas states.

Here, we tested whether bladder cancer contains a recurrent CAF/TGF-beta/endothelial barrier direction associated with immune exclusion and checkpoint resistance. We reconstructed a discovery representation in GSE171351 and assessed recurrence of both projected components and a direct module-based barrier score in the independent MIBC spatial atlas GSE319536 [8,20]. We then projected the prespecified scores into TCGA-BLCA, IMvigor210, GSE176307 and GSE328930 to evaluate prognosis-oriented and ICI-response associations. The analytic frame distinguishes a spatial barrier core from a heuristic barrier-exclusion score used as a transparent, hypothesis-generating clinical translation axis; neither score is presented as establishing causality or a clinically validated biomarker.

## Materials and methods

### Study design and public data sources

We performed a retrospective, public-data bioinformatics study using de-identified bladder or urothelial cancer datasets. The workflow proceeded through prespecified analysis layers: dataset-readability checks, signature scoring, spatial NMF discovery, direct spatial recurrence testing, bulk clinical projection, external ICI projection and reviewer-focused sensitivity analyses. The primary spatial feature was the CAF/TGF-beta/endothelial barrier direction. The clinical translation score was a locked barrier-exclusion score that incorporated barrier activation and reduced CD8 cytotoxic and tertiary lymphoid structure (TLS)/B-cell immune accessibility.

GSE171351 served as the spatial discovery cohort and included 4 Visium sections and 4,086 spots [8]. GSE319536 served as the external spatial recurrence cohort and included 22 Visium sections and 103,011 processed spots in the present pipeline [20]. TCGA-BLCA was used for prognosis-oriented bulk support [7]. IMvigor210 was the main anti-PD-L1 clinical response cohort [3,4,6]. GSE176307 provided an external real-world ICI-treated urothelial carcinoma cohort [21]. GSE328930/DUTRENEO was retained only as exploratory MIBC treatment-response evidence because of its small sample size and distinct neoadjuvant setting [22]. No other datasets were included in the reported analyses (Tables 1 and 2).

**Table 1:** Public datasets, platforms, analysis sizes, roles and limitations.

Cohort	Data type	Disease setting	Platform	Analysis size	Treatment context	Primary role	Key limitation
GSE171351	Spatial transcriptomics	Treatment-naive MIBC	10x Visium	4 sections; 4,086 spots	None in the spatial sections	Discovery of the rank-5 NMF representation and direct CAF/TGF-beta/endothelial barrier direction	Only four sections; dominant NMF assignment was strongly associated with section identity; no ICI endpoint
GSE319536	Spatial transcriptomics	MIBC	10x Visium	22 sections; 103,011 processed spots	Not used as a treatment-response cohort	External recurrence testing of the direct barrier direction and projected components	The discovery E2/E4 separation did not reproduce; no patient-level ICI response endpoint was analyzed
TCGA-BLCA	Bulk RNA-seq and clinical	Bladder urothelial carcinoma	RNA-seq	406 RNA-seq cases; 376 with overall-survival analysis data	Predominantly non-ICI primary tumors	Prognosis-oriented support and continuous-versus-top-quartile sensitivity analysis	Bulk data lack spatial coordinates and do not provide an ICI response endpoint
IMvigor210	Bulk RNA-seq and clinical	Metastatic urothelial carcinoma	RNA-seq	298 response-evaluable; 205 in the full covariate model	Atezolizumab	Primary anti-PD-L1 response projection and model-comparison cohort	Metastatic setting; the fully adjusted E2-high estimate was borderline and discrimination gain was small
GSE176307	Bulk RNA-seq and clinical	Metastatic urothelial carcinoma	Ion Torrent RNA-seq	88 response-evaluable samples	Mixed real-world ICI agents	Independent real-world ICI response projection	Small event counts and mixed agents; not a spatial validation cohort
GSE328930/DUTRENEO	Bulk expression with response metadata	MIBC	Expression profiling	28 HOT-ICI samples used in the ICI analysis	Neoadjuvant ICI arm	Exploratory small-cohort sensitivity analysis	Small neoadjuvant cohort; therefore excluded in a dedicated pooled sensitivity analysis

Abbreviations are defined in the manuscript and S1 Table.

**Table 2:** Variables actually analyzed and their harmonization rules.

Variable	Definition	Cohorts	Harmonization rule	Missing-data action	Actual use
cohort_id	Public study identifier	All six cohorts	Analyze each cohort separately	No cross-platform raw-expression pooling	All analyses
sample_id	Original sample or spatial-section identifier	All six cohorts	Preserve the source identifier and create a normalized audit identifier	Do not merge without an explicit mapping	All analyses and overlap audit
patient_id	Patient-level identifier when supplied	TCGA-BLCA; IMvigor210; GSE176307; GSE328930	Keep sample and patient identifiers separate	Treat samples as independent only when patient mapping is unavailable and state the limitation	Clinical models and overlap audit
expression_matrix	Gene-expression matrix	All six cohorts	Upper-case canonical gene symbols; collapse duplicate features as specified by platform	Compute a reduced module only when coverage is recorded	Module scoring
spatial_coordinates	Within-section spot coordinates	GSE171351; GSE319536	Use section-specific Euclidean coordinates; never compare absolute coordinates across sections	Downgrade to sample-level analysis if absent	Neighborhood sensitivity and spatial recurrence
module_scores	Eleven locked expression modules	All six cohorts	Mean normalized expression of available genes followed by within-cohort standardization	Report requested/found genes in Supplementary S1 Table	All mechanism and clinical scores
NMF_component_scores	Rank-5 spatial component scores	GSE171351; projected in GSE319536 and selected bulk sensitivity analyses	Use the submitted 28-feature transformation; retain rank 5 as prespecified rather than uniquely optimal	Do not substitute a new rank in primary results	Discovery representation and projection sensitivity
barrier_core_score	Standardized mean of CAF/ECM, TGF-beta/EMT and endothelial-remodeling modules	All six cohorts	Compute within cohort	Not calculable when a required module is unavailable	Primary spatial mechanism axis
barrier_exclusion_score	z(barrier core – 0.50 x CD8 cytotoxicity – 0.25 x TLS/B-cell)	All six cohorts	Coefficients were fixed before response-cohort analysis and were never tuned or refit	Do not replace with an optimized alternative	Hypothesis-generating clinical translation axis
response	Clinical or pathological treatment response	IMvigor210; GSE176307; GSE328930	CR/PR or source-defined response versus non-response; preserve original labels	Exclude records lacking a response label	Logistic models and meta-analysis
overall_survival	Overall-survival time and event	TCGA-BLCA	Use source time/event definitions and complete cases	No imputation	Cox models
TMB	Tumor mutational burden	IMvigor210	Use the source measure in covariate-adjusted models	Complete-case model	Adjusted response analysis
immune_phenotype	Source immune phenotype	IMvigor210	Retain source categories	Complete-case model	Adjusted response analysis
TCGA_subtype	Source molecular subtype	IMvigor210	Retain source categories	Complete-case model	Adjusted response analysis
PD-L1_IC_TC	PD-L1 immune-cell and tumor-cell categories	IMvigor210	Keep IC and TC categories as source covariates	Complete-case model	Adjusted response analysis
age_sex_stage	Clinical survival covariates	TCGA-BLCA	Use complete age, sex and pathological-stage records	Complete-case model	Adjusted survival analysis

Abbreviations are defined in the manuscript and S1 Table.

### Data preprocessing and endpoint harmonization

Spatial and bulk expression matrices were processed separately within each cohort. Gene symbols were upper-cased and canonicalized before signature matching. When platform-specific processing required one value per gene, duplicated gene symbols were collapsed by retaining the feature with the highest mean expression. Count-based sparse spatial matrices were library-size normalized to 10,000 counts per spot and log1p transformed. Dense bulk count matrices were library-size normalized to 1,000,000 counts per sample and log1p transformed. H5AD and matrix files were handled with Python-based single-cell and spatial-analysis tooling using AnnData-compatible data structures [23].

Clinical endpoints were harmonized within each cohort before modeling. In IMvigor210, complete and partial responses were coded as response, and stable or progressive disease was coded as non-response. In GSE176307 and GSE328930, response labels were taken from cleaned metadata generated during dataset-readability checks. TCGA-BLCA overall survival was analyzed using available survival time and vital status. Complete-case analysis was used for each fitted model, and the corresponding sample size was reported for each model class.

### Signature definitions and module scoring

Gene signatures were locked before manuscript writing. Modules covered epithelial tumor, CAF/extracellular matrix (ECM), hypoxia, TGF-beta/epithelial-mesenchymal transition (EMT), endothelial remodeling, myeloid suppressive, CD8 cytotoxic, TLS/B-cell, checkpoint PD1/PD-L1, mast-cell and proliferation programs. For each cohort, module scores were calculated as the average log-normalized expression of available genes in the module and then z-scored within cohort. Exact gene symbols, module sizes, pairwise overlaps and cohort-specific gene coverage are reported in S1 Table. The 11 modules contained 117 unique genes; CAF/ECM and TGF-beta/EMT shared COL1A1 and COL3A1, while all other module pairs had no overlapping genes. Gene coverage was recorded so that reduced signatures could be distinguished from complete signatures.

An exploratory stromal-hypoxic seed score was first computed as the mean of CAF/ECM_z, Hypoxia_z, TGF-beta/EMT_z, Endothelial_Remodeling_z and Myeloid_Suppressive_z. An exploratory immune-exclusion seed score was defined as the stromal-hypoxic seed score minus 0.5 times CD8_Cytotoxic_z. These seed scores initiated spatial reconstruction but were superseded in the final manuscript by the CAF/TGF-beta/endothelial barrier core and barrier-exclusion axes.

### Spatial-neighborhood features and non-negative matrix factorization discovery

In GSE171351, spatial-neighborhood features were calculated within each section using Euclidean spot coordinates. For each spot, the six nearest neighboring spots were identified with a k-d tree, excluding the index spot. Six neighbors were retained as the prespecified local summary because it approximates the immediate Visium lattice neighborhood while remaining applicable to sections with incomplete capture geometry. Neighbor-average module scores were then calculated for each signature. A spatial-context exclusion score was defined as the mean of own stromal arms plus the mean of neighboring stromal arms, minus 0.5 times neighboring CD8_Cytotoxic_z and 0.25 times neighboring TLS_Bcell_z. Sensitivity analyses repeated the score with k = 4, 8 and 12 and with a radius graph defined by each section’s median sixth-neighbor distance.

The non-negative matrix factorization (NMF) input matrix included own module z-scores, neighbor module z-scores and derived context scores. Features were standardized, clipped to the range of −4 to +4, shifted to non-negative values and decomposed using rank-5 NMF with 30 random initializations and multiplicative updates [24]. The best solution was selected by Frobenius reconstruction error. Rank sensitivity compared ranks 3–7 with 30 initializations per rank. Consensus cophenetic correlation, dispersion, proportion of ambiguous clustering (PAC), matched-component cosine similarity and association between dominant component and section identity were calculated. Rank 5 was retained as the prespecified discovery representation rather than claimed to be uniquely optimal. Component loadings were normalized, and the component with maximal stromal-context enrichment and immune-access penalty was selected as the initial stromal barrier candidate. High-state spots were defined by top-quartile component scores.

### E2/E4 discovery components and portable score definitions

The selected E2 component was described as barrier enriched because of its CAF/ECM, TGF-beta/EMT and endothelial-remodeling loadings. E4 was described as a hypoxic epithelial component. E2-only, E4-only and dual-high states were defined with top-quartile component thresholds in GSE171351. E2/E4 spatial overlap, Spearman correlation and high-state module contrasts were used to characterize their relationship within the discovery cohort; external analyses were required before any cross-cohort interpretation.

For portable scoring, the E2 barrier-core score was defined as the within-cohort z-scored mean of CAF_ECM_z, TGFb_EMT_z and Endothelial_Remodeling_z. The E4 hypoxic epithelial score was defined as the within-cohort z-scored mean of Hypoxia_z and Epithelial_Tumor_z. The barrier-exclusion score was defined as:

z(E2_barrier_core_score - 0.50 * CD8_Cytotoxic_z − 0.25 * TLS_Bcell_z)

The coefficients 0.50 and 0.25 are heuristic rather than data-driven. They were carried forward unchanged from the spatial-context exclusion definition, fixed before any response-cohort analysis, and never tuned, optimized or refit in IMvigor210, GSE176307 or GSE328930. A coefficient-grid sensitivity analysis was used only to assess robustness and was not used to select a preferred score.

An E2/E4 composite score was defined as the z-scored mean of E2_barrier_core_score and E4_hypoxic_epithelial_companion_score. High states for these axes were defined using cohort-specific top-quartile thresholds.

### External spatial recurrence analysis

External recurrence was assessed in GSE319536 using both projected E2/E4 component scores and direct module-based barrier and hypoxic epithelial scores [20]. Component projection applied the GSE171351-derived feature transformation and NMF weights to the external spatial module-score matrix. Direct recurrence used the CAF/TGF-beta/endothelial barrier score and hypoxic epithelial score without relying on NMF projection. Within-sample top-quartile analyses were prioritized over global high-state fractions because spatial sections differed in baseline composition. Sample-adjusted Pearson correlations were estimated after residualizing module scores against sample identity. Spearman correlations were used for global and within-sample score associations.

### Bulk projection and clinical modeling

For bulk cohorts, module scores were computed using the same locked signature definitions and within-cohort z-scoring. When NMF-derived spatial features were projected to bulk data, neighbor features were approximated by the corresponding sample-level module z-scores, and the GSE171351-derived transformation parameters and NMF weights were used to obtain projected component scores. The transparent module-based barrier core and barrier-exclusion scores were emphasized because they are more portable and interpretable than spatial-neighbor approximations in bulk data.

Clinical covariates were included when available. In IMvigor210, covariate-adjusted models included tumor mutational burden (TMB), immune phenotype, TCGA subtype and PD-L1 IC/TC levels [3,4,6]. In TCGA-BLCA, clinical survival models adjusted for age, sex and pathological stage [7]. In GSE176307 and GSE328930, covariate-adjusted models used available cleaned metadata when sample size and event counts supported model fitting [21,22]. TCGA-BLCA, IMvigor210, GSE176307 and GSE328930 originated from separate source studies, and no duplicate normalized public sample or patient identifiers were detected across the six pairwise cohort comparisons. Direct cross-study identity linkage was not possible because the public identifiers were independently anonymized.

### Statistical analysis

Continuous module and component scores were z-scored within cohort before modeling unless otherwise specified. Group comparisons of spatial module scores used Wilcoxon rank-sum tests, and multiple testing across module comparisons was controlled by the Benjamini-Hochberg false discovery rate procedure. Associations between continuous spatial scores and modules were assessed with Spearman correlation. Categorical enrichment analyses used Fisher exact tests or chi-square tests where appropriate.

Treatment-response associations were evaluated with logistic regression. Effect estimates are reported as odds ratios (ORs) with 95% confidence intervals (CIs). Survival associations were evaluated with Cox proportional hazards regression and reported as hazard ratios (HRs) with 95% CIs. Binary model discrimination was summarized using the area under the receiver operating characteristic curve (AUC); receiver operating characteristic analyses were implemented with pROC where applicable [25]. Nested logistic models were compared using likelihood-ratio tests, delta Akaike information criterion and delta AUC. Response effects across ICI cohorts were summarized by inverse-variance fixed-effect models and restricted maximum-likelihood random-effects models on log ORs. Heterogeneity was summarized using I2 and tau-squared. Pooled analyses were designated exploratory because only two or three heterogeneous cohorts were available. A dedicated sensitivity analysis excluded GSE328930. All p values were two-sided.

### Reviewer-focused sensitivity analyses

To test whether the barrier-exclusion score was merely a low-CD8/TLS surrogate, response models were compared across four prespecified model classes: barrier-core-only, CD8/TLS-only, core-plus-CD8/TLS and barrier-exclusion. These comparisons were performed in IMvigor210, GSE176307 and the GSE328930 HOT-ICI subset and are reported separately from the pooled analyses (Tables 5 and 6). Additional revision analyses comprised NMF rank and initialization stability, alternative neighborhood definitions, a 12-combination coefficient grid, fixed- and random-effects meta-analysis with and without GSE328930, and the cross-cohort identifier audit. These analyses were used to test robustness and define evidence strength, not to tune the clinical score or establish a universal ecotype architecture.

### Software, reproducibility and ethics

Analyses were performed with custom Python and R scripts. Python 3.10.11 was used with pandas 2.2.2, numpy 1.26.4, scipy 1.15.3, anndata 0.11.4 and matplotlib 3.10.8 for matrix handling, signature scoring, spatial-neighborhood construction, NMF projection and visualization [23,24]. R 4.5.3 was used with pROC 1.19.0.1, ggplot2 4.0.2 and survival 3.8.6 for logistic regression, Cox regression, AUC calculation and visualization [25]. Manuscript and table DOCX exports used python-docx 1.2.0. The revision scripts for NMF stability, neighborhood sensitivity, weight/meta-analysis sensitivity, cohort-overlap audit and signature-table preparation are included with the original analysis scripts in the rebuilt S1 File. Key tabular outputs are reported in Tables 1-6 and S1 Table.

This study used publicly available, de-identified transcriptomic and clinical datasets and did not involve new patient recruitment or intervention. All source datasets were accessed for research purposes and finalized for this analysis on 27 May 2026. The authors had no access to information that could identify individual participants during or after data collection. Local institutional review board approval was not required for this secondary analysis. Source datasets are listed in Table 1. Analysis scripts and key processed outputs are provided in S1 File for peer-review access and reproducibility.

## Results

### Cohort architecture and analysis overview

**Fig 1:**
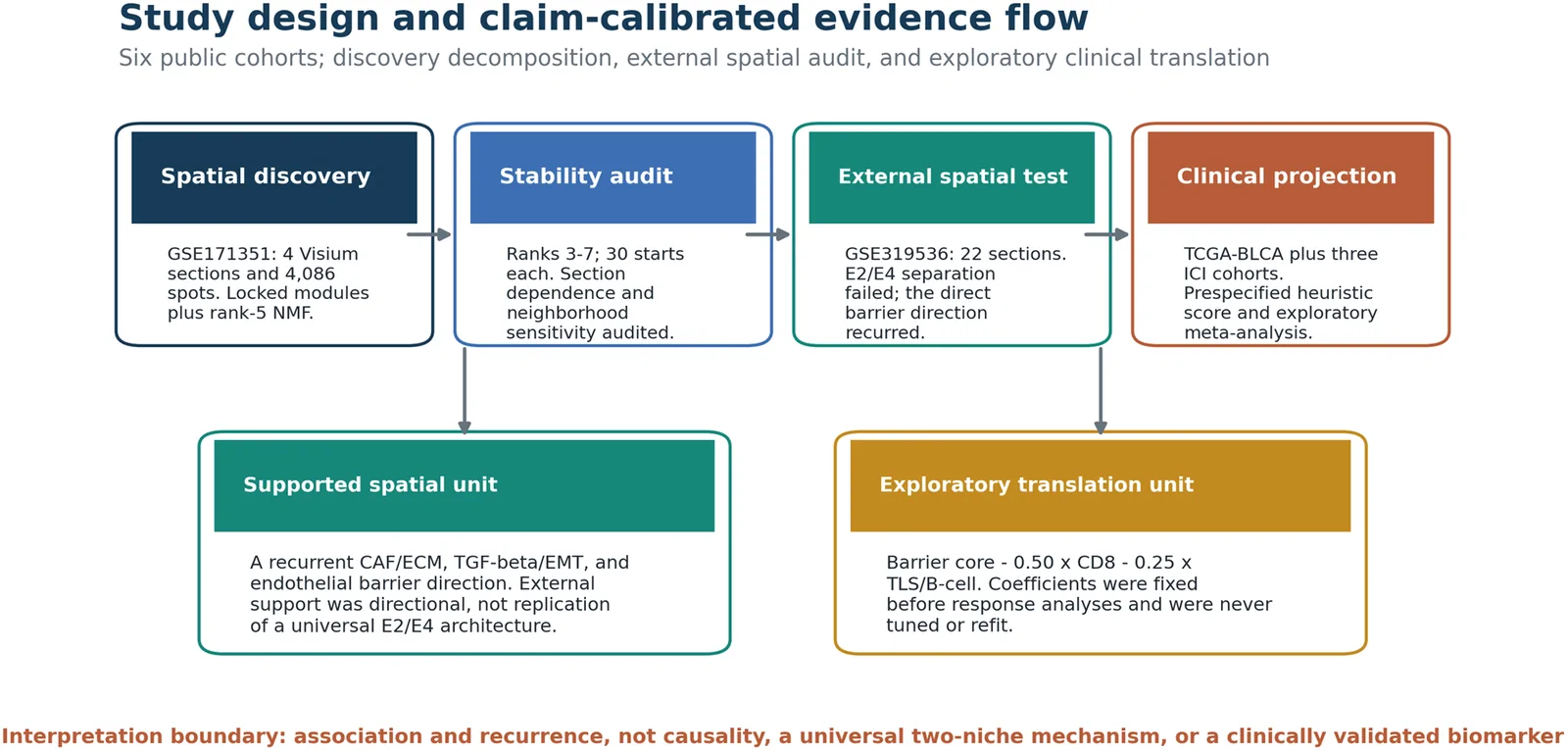
Study design and spatial-to-clinical analysis workflow. Six public cohorts were organized into spatial discovery, external spatial recurrence, prognosis-oriented support and treatment-response projection. The final model distinguishes a recurrent CAF/TGF-beta/endothelial barrier direction from a heuristic barrier-exclusion translation score.

We assembled six public cohorts to examine whether spatially organized stromal and vascular programs were associated with immune exclusion and checkpoint resistance in bladder cancer. Spatial discovery used GSE171351 (4 sections; 4,086 spots) [8]. Independent recurrence testing used GSE319536 (22 sections; 103,011 processed spots in the present pipeline) [20]. Bulk analyses used TCGA-BLCA for prognosis-oriented support [7], IMvigor210 for primary anti-PD-L1 response projection [3,4,6], GSE176307 for external real-world ICI projection [21], and GSE328930/DUTRENEO as an exploratory small-cohort sensitivity analysis [22] (Fig 1; Tables 1 and 2).

The locked analysis comprised 11 modules representing epithelial tumor, CAF/ECM, hypoxia, TGF-beta/EMT, endothelial remodeling, myeloid suppressive, CD8 cytotoxic, TLS/B-cell, checkpoint, mast-cell and proliferation programs. The exact 117 unique genes, module sizes, pairwise overlaps and cohort-specific coverage are reported in S1 Table. The four clinical cohorts originated from separate source studies, and no duplicate normalized public sample or patient identifiers were detected across the six pairwise comparisons. Because identifiers were independently anonymized, direct patient-identity linkage across studies was not possible (Table 6).

### Discovery NMF identifies separable barrier-enriched and hypoxic epithelial components

In GSE171351, spot-level module scores were augmented with within-section neighborhood features and decomposed with the prespecified rank-5 NMF [24]. Across 30 rank-5 initializations, the consensus cophenetic correlation was 0.990, PAC was 0.123, consensus dispersion was 0.893 and the median matched-component cosine similarity was 0.960. Ranks 3–7 showed the expected reconstruction-versus-complexity trade-off; rank 5 was retained as the submitted discovery representation rather than as a uniquely optimal solution. Dominant rank-5 component assignment remained strongly associated with section identity (Cramer’s V = 0.944), demonstrating that the four-section design substantially influenced the NMF architecture (S1 Fig; Table 3).

**Table 3:** Spatial discovery, external non-replication and robustness audit.

Layer	Diagnostic	Estimate	Support	Verification file	Interpretation
GSE171351 NMF	Input dimensions	4,086 spots; 28 non-negative features	Ranks 3–7; 30 initializations per rank	S1 File/analysis/nmf/nmf_stability_report.md	Small discovery set; results require sample-aware and external checks
GSE171351 NMF	Rank-5 stability	Cophenetic r = 0.990; PAC = 0.123; dispersion = 0.893	Median matched-component cosine = 0.960	S1 File/analysis/nmf/nmf_rank_stability_summary.csv	Rank 5 was reproducible but is retained as prespecified, not uniquely optimal
GSE171351 NMF	Section association	Dominant-component Cramer’s V = 0.944	Best rank-5 run	S1 File/analysis/nmf/nmf_rank_stability_summary.csv	Section identity substantially influenced the NMF architecture
GSE171351 E2/E4	Discovery high-state separation	E2-only = 1,019; E4-only = 1,019; dual-high = 3; rho = −0.375	4,086 spots	S1 File/analysis/spatial/spatial_replication_sensitivity_report.md	A discovery-cohort pattern, not a universal two-niche architecture
GSE171351 E2/E4	High-state versus section identity	Cramer’s V = 0.660	Four sections	S1 File/analysis/spatial/spatial_replication_sensitivity_report.md	Global high-state fractions are potentially sample confounded
GSE171351 neighborhood	Agreement with primary k = 6 score	Median rho: k4 = 0.973; k8 = 0.985; k12 = 0.972; radius = 0.985	Four sections	S1 File/analysis/neighborhood/neighborhood_score_agreement_summary.csv	Spot-level barrier score was robust to alternative local-neighborhood definitions
GSE171351 neighborhood	Barrier-direction preservation	CAF/ECM, TGF-beta/EMT and endothelial directions positive in 4/4 sections for every method	k = 4,6,8,12 and radius graph	S1 File/analysis/neighborhood/neighborhood_correlation_summary.csv	Primary barrier direction did not depend on k = 6
GSE319536 recurrence	Projected E2/E4 relationship	Spearman rho = 0.928	22 spatial sections	S1 File/analysis/spatial/spatial_replication_sensitivity_report.md	The discovery E2/E4 mutual exclusivity did not reproduce
GSE319536 recurrence	Direct barrier/hypoxic relationship	Spearman rho = 0.382; sample-adjusted Pearson r = 0.230	103,011 processed spots	S1 File/analysis/spatial/spatial_replication_sensitivity_report.md	Barrier and hypoxic epithelial programs can co-occur in the external cohort
GSE319536 recurrence	Direct barrier direction	Expected CAF/ECM, TGF-beta/EMT and endothelial direction in 22/22 sections	Within-section analyses	S1 File/analysis/spatial/spatial_replication_sensitivity_report.md	External evidence supports a recurrent barrier direction, not a universal two-niche claim
GSE319536 neighborhood	Agreement with primary k = 6 score	Median rho: k4 = 0.983; k8 = 0.990; k12 = 0.981; radius = 0.993	22 sections	S1 File/analysis/neighborhood/neighborhood_score_agreement_summary.csv	External barrier scores were robust to neighborhood definition
GSE319536 neighborhood	Barrier-direction preservation	CAF/ECM, TGF-beta/EMT and endothelial directions positive in 22/22 sections for every method	k = 4,6,8,12 and radius graph	S1 File/analysis/neighborhood/neighborhood_correlation_summary.csv	External recurrence was not an artifact of the k = 6 choice

Abbreviations are defined in the manuscript and S1 Table. Verification paths identify the corresponding archived output within S1 File.

**Fig 2:**
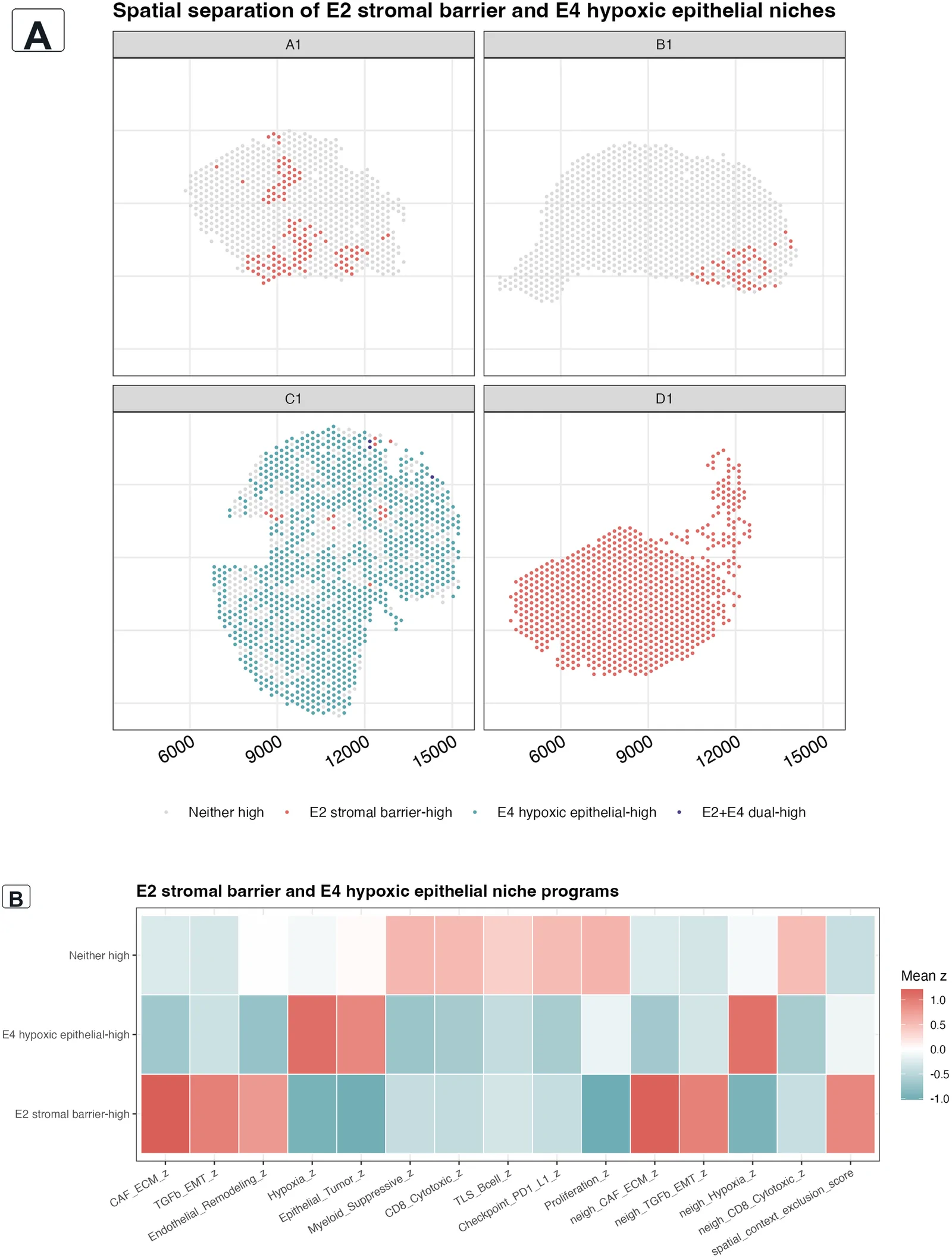
Discovery-cohort E2 and E4 components in GSE171351. Rank-5 NMF separated an E2 barrier-enriched component from an E4 hypoxic epithelial component within four sections. The near-disjoint top-quartile pattern is presented as a discovery-cohort decomposition because component assignment was strongly associated with section identity and the separation did not reproduce externally.

Within the rank-5 representation, E2 carried the strongest stromal-context pattern, including CAF/ECM, TGF-beta/EMT, endothelial remodeling and their neighborhood features. E4 was enriched for hypoxia, epithelial tumor and their neighborhood features. Top-quartile states were almost disjoint within GSE171351: 1,019 spots were E2-only high, 1,019 were E4-only high and 3 were dual-high; E2 and E4 were negatively correlated (Spearman rho = −0.375). Because this pattern arose from only four sections and high-state assignment was associated with sample identity (Cramer’s V = 0.660), it is treated as a discovery-cohort decomposition, not a universal two-niche architecture (Fig 2; Table 3).

### E2 is enriched for a CAF/TGF-beta/endothelial barrier core in the discovery cohort

Compared with E4-only high spots, E2-only high spots showed higher CAF/ECM (mean difference = 1.89), TGF-beta/EMT (1.34) and endothelial-remodeling (1.54) scores and lower hypoxia (−2.05) and epithelial-tumor (−1.92) scores. In the broader E2-high versus E2-low comparison, E2-high spots also showed higher CAF/ECM (1.61), TGF-beta/EMT (1.30) and endothelial-remodeling (1.04) scores. E2 was therefore interpreted as a barrier-enriched discovery component rather than as a hypoxic tumor state.

### External spatial data reproduce the barrier direction but not E2/E4 separation

Sample-aware analyses first tested whether the GSE171351 result depended on a single neighborhood definition. For k = 4, 6, 8 and 12 and a radius graph defined from the median sixth-neighbor distance, CAF/ECM, TGF-beta/EMT and endothelial associations were positive in 4 of 4 sections. Agreement with the primary k = 6 barrier score was high (median rho = 0.972–0.985 across alternatives; S2 Fig; Table 3).

**Fig 3:**
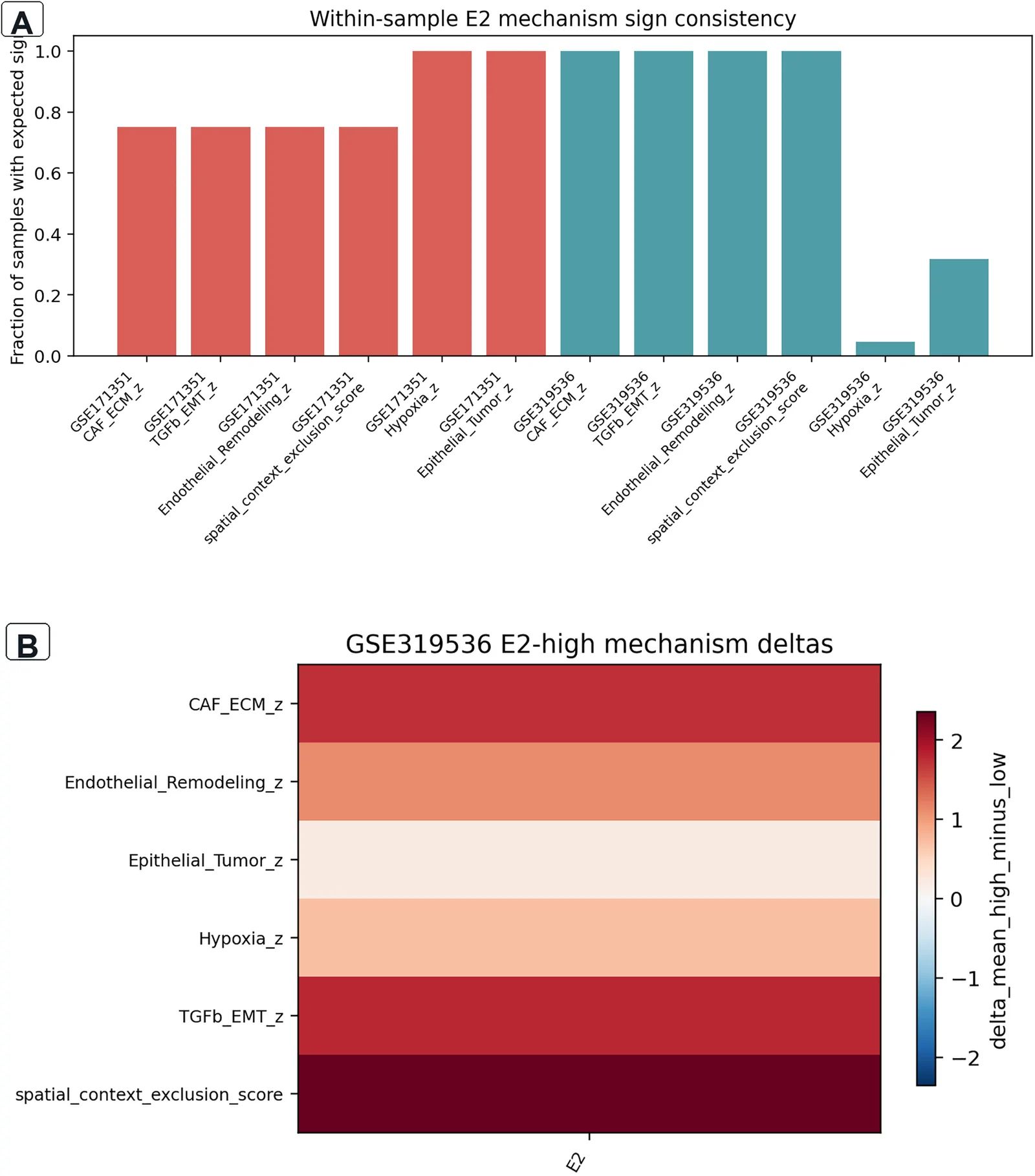
External spatial recurrence of the barrier direction and non-replication of E2/E4 separation. GSE319536 preserved the direct CAF/ECM, TGF-beta/EMT and endothelial direction in 22 of 22 sections, while projected E2 and E4 were positively correlated. The replicated finding is the barrier direction rather than a universal two-niche architecture.

In GSE319536, the projected E2 component and direct CAF/TGF-beta/endothelial score showed the expected barrier direction in 22 of 22 sections [20]. This result was preserved across all five neighborhood definitions, with median score agreement against k = 6 of rho = 0.981–0.993. However, the discovery E2/E4 mutual exclusivity did not reproduce. Projected E2 and E4 scores were strongly positively correlated (Spearman rho = 0.928), and direct barrier and hypoxic epithelial scores also co-varied (Spearman rho = 0.382; sample-adjusted Pearson r = 0.230). Thus, the replicated finding is a recurrent barrier direction; the relationship between the barrier and hypoxic epithelial programs is cohort dependent (Fig 3; S2 Fig; Table 3).

### E2-high shows suggestive evidence of anti-PD-L1 non-response in IMvigor210

**Fig 4:**
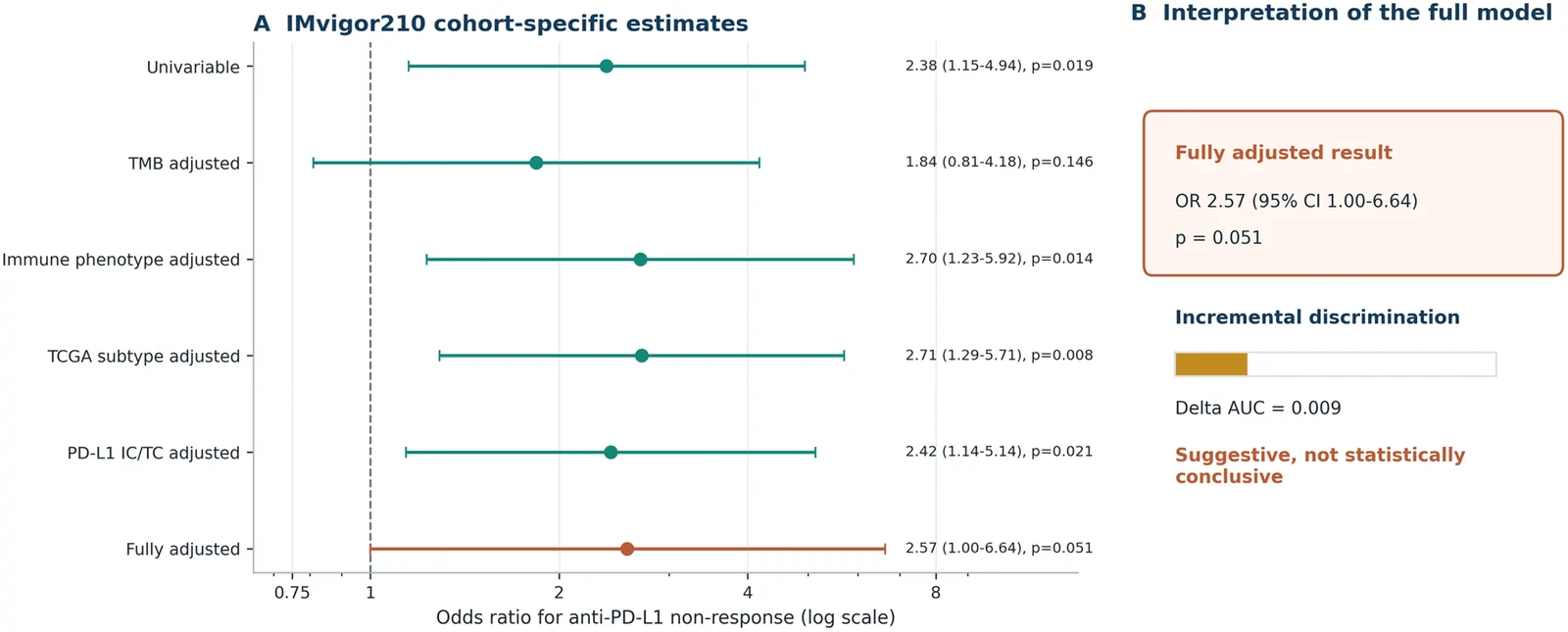
IMvigor210 E2-high response models. Forest and response-summary panels show cohort-specific univariable and covariate-adjusted estimates. The fully adjusted estimate was imprecise (p = 0.051) and the delta AUC was 0.009; the result is therefore interpreted as suggestive rather than conclusive.

Among 298 response-evaluable IMvigor210 patients, 230 were non-responders. Top-quartile E2-high tumors showed increased odds of non-response in the univariable model (OR=2.38, 95% CI 1.15–4.94, p = 0.019). In the full biologic covariate model, the estimate was imprecise and did not cross the conventional significance threshold (OR=2.57, 95% CI 1.00–6.64, p = 0.051). The discrimination gain was small (delta AUC = 0.009), despite a likelihood-ratio p value of 0.041. The fully adjusted result is therefore described as suggestive but not statistically conclusive, and E2-high is not presented as an independently validated response biomarker (Fig 4; Table 4).

**Table 4:** Primary cohort-specific IMvigor210 and TCGA-BLCA clinical models.

Cohort	Endpoint	Model	Predictor	N	Events	Effect	Estimate (95% CI)	P value	Interpretation
IMvigor210	anti-PD-L1 non-response	univariable	E2 stromal barrier high	298	230	OR	2.38 (1.15-4.94)	0.019	Supportive association; cohort-specific estimate
IMvigor210	anti-PD-L1 non-response	TMB-adjusted	E2 stromal barrier high	234	173	OR	1.84 (0.81-4.18)	0.146	Supportive association; cohort-specific estimate
IMvigor210	anti-PD-L1 non-response	immune phenotype-adjusted	E2 stromal barrier high	244	183	OR	2.70 (1.23-5.92)	0.014	Supportive association; cohort-specific estimate
IMvigor210	anti-PD-L1 non-response	TCGA subtype-adjusted	E2 stromal barrier high	298	230	OR	2.71 (1.29-5.71)	0.008	Supportive association; cohort-specific estimate
IMvigor210	anti-PD-L1 non-response	PD-L1 IC/TC-adjusted	E2 stromal barrier high	297	229	OR	2.42 (1.14-5.14)	0.021	Supportive association; cohort-specific estimate
IMvigor210	anti-PD-L1 non-response	full biologic covariates	E2 stromal barrier high	205	151	OR	2.57 (1.00-6.64)	0.051	Suggestive but not statistically conclusive after full adjustment; delta AUC was 0.009
TCGA-BLCA	overall survival	univariable continuous per SD	E2 stromal barrier score	376	177	HR	1.22 (1.05-1.42)	0.009	Continuous adverse-risk support; not proof of an independent prognostic biomarker
TCGA-BLCA	overall survival	univariable top quartile	E2 stromal barrier high	376	177	HR	1.23 (0.89-1.70)	0.202	Thresholded effect was not significant; dichotomization reduced information and power
TCGA-BLCA	overall survival	clinical multivariable top quartile	E2 stromal barrier high	370	172	HR	1.08 (0.78-1.51)	0.633	Thresholded effect was not significant; dichotomization reduced information and power

Abbreviations are defined in the manuscript and S1 Table.

### An exploratory E2/E4 composite is retained only as a sensitivity axis

Because E2 and E4 separated only in the discovery cohort, their composite was evaluated as an exploratory response sensitivity axis rather than as a biological ecotype. In IMvigor210, the composite-high state showed increased odds of non-response in the full covariate model (OR=3.25, 95% CI 1.16–9.09, p = 0.025; delta AUC = 0.023). The external positive E2/E4 correlation and discovery-cohort sample dependence preclude interpreting this result as validation of a universal two-niche mechanism.

### TCGA-BLCA supports a continuous adverse-risk association but not a prognostic threshold

**Fig 5:**
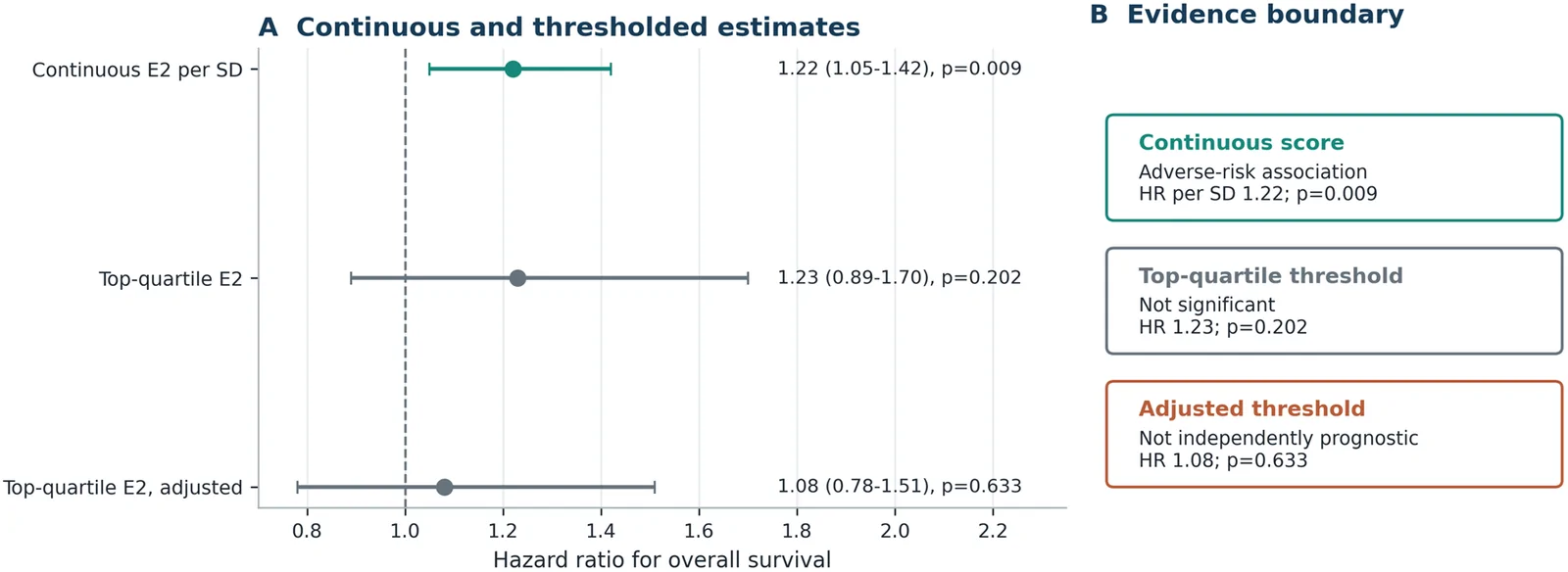
TCGA-BLCA continuous and thresholded survival analyses. Continuous E2 was associated with overall survival, whereas top-quartile E2-high was not significant after clinical adjustment. The figure contrasts continuous adverse-risk support with the absence of an independently prognostic threshold.

In 376 TCGA-BLCA patients with overall-survival data, continuous E2 was associated with mortality risk in univariable Cox regression (HR per standard deviation = 1.22, 95% CI 1.05–1.42, p = 0.009) [7]. The top-quartile E2-high state was not significant after adjustment for age, sex and pathological stage (n = 370; events = 172; HR = 1.08, p = 0.633). The discrepancy is compatible with information loss and reduced power after dichotomizing a continuous, potentially non-linear risk gradient; it does not support an independently prognostic threshold. TCGA-BLCA therefore provides continuous adverse-risk support only (Fig 5; Table 4).

### The heuristic barrier-exclusion score shows exploratory cross-cohort support

The pure barrier core was weak in IMvigor210 (OR per standard deviation = 1.06, p = 0.649) and non-significant in GSE176307 (OR=1.46, p = 0.186) and the GSE328930 HOT-ICI subset (OR=1.36, p = 0.438). Its three-cohort fixed-effect summary was also non-significant (OR=1.14, 95% CI 0.91–1.44, p = 0.246; I2 = 0%). The barrier core is therefore interpreted as a spatial-mechanistic score, not a standalone clinical response biomarker.

**Fig 6:**
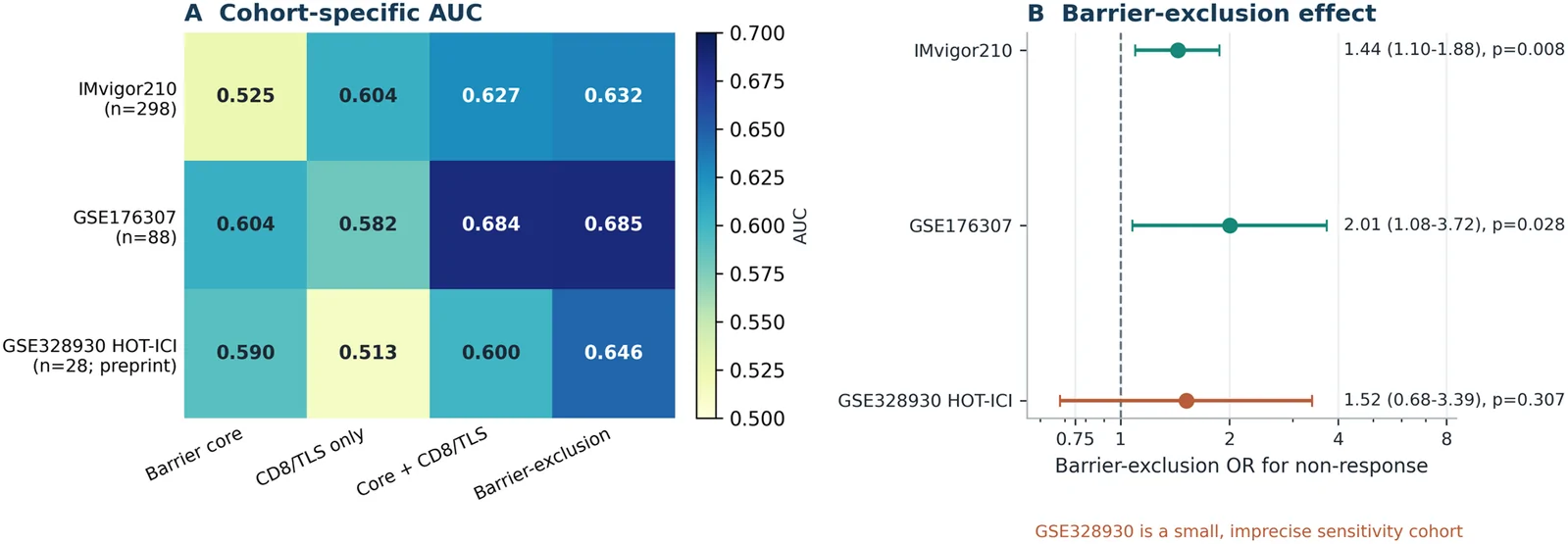
Single-cohort barrier and immune-access model comparisons. Cohort-specific AUCs and effect estimates are shown separately from the pooled meta-analysis. The 28-patient GSE328930 HOT-ICI result is labeled as an imprecise small-cohort sensitivity estimate.

The heuristic barrier-exclusion score showed stronger cohort-specific effects in IMvigor210 (OR=1.44, 95% CI 1.10–1.88, p = 0.008) and GSE176307 (OR=2.01, 95% CI 1.08–3.72, p = 0.028), whereas the 28-patient GSE328930 HOT-ICI estimate was imprecise and non-significant (OR=1.52, 95% CI 0.68–3.39, p = 0.307). The exploratory three-cohort fixed-effect and REML random-effects summaries were nearly identical (OR=1.52, 95% CI 1.20–1.92; I2 = 0%; tau-squared approximately 0). Excluding GSE328930 yielded OR=1.52 (95% CI 1.19–1.94). With only two or three heterogeneous cohorts, low I2 does not establish homogeneity; these pooled findings are supportive rather than confirmatory (Fig 6; Tables 5 and 6).

**Table 5:** Single-cohort response-model comparisons.

Cohort	Endpoint	Model	N	Events	AUC	AIC	Predictor effect	Interpretation
IMvigor210	anti-PD-L1 non-response	barrier_core_only	298	230	0.525	323.9	barrier core: OR 1.06 (0.82–1.38), p = 0.649	Cohort-specific exploratory model comparison
IMvigor210	anti-PD-L1 non-response	cd8_tls_only	298	230	0.604	318.7	CD8/TLS immune access: OR 0.73 (0.56–0.96), p = 0.022	Cohort-specific exploratory model comparison
IMvigor210	anti-PD-L1 non-response	core_plus_cd8_tls	298	230	0.627	316.4	barrier core: OR 1.40 (1.02-1.91), p=0.038; CD8/TLS immune access: OR 0.61 (0.44-0.84), p=0.003	Cohort-specific exploratory model comparison
IMvigor210	anti-PD-L1 non-response	barrier_exclusion_score	298	230	0.632	316.9	barrier-exclusion score: OR 1.44 (1.10–1.88), p = 0.008	Cohort-specific exploratory model comparison
GSE176307	real-world ICI non-response	barrier_core_only	88	72	0.604	85.6	barrier core: OR 1.46 (0.83–2.55), p = 0.186	Cohort-specific exploratory model comparison
GSE176307	real-world ICI non-response	cd8_tls_only	88	72	0.582	86.4	CD8/TLS immune access: OR 0.75 (0.43–1.31), p = 0.314	Cohort-specific exploratory model comparison
GSE176307	real-world ICI non-response	core_plus_cd8_tls	88	72	0.684	84.3	barrier core: OR 1.96 (0.99-3.90), p=0.054; CD8/TLS immune access: OR 0.56 (0.30-1.05), p=0.072	Cohort-specific exploratory model comparison
GSE176307	real-world ICI non-response	barrier_exclusion_score	88	72	0.685	82.0	barrier-exclusion score: OR 2.01 (1.08–3.72), p = 0.028	Cohort-specific exploratory model comparison
GSE328930 HOT-ICI	ICI pathologic non-response	barrier_core_only	28	13	0.590	42.1	barrier core: OR 1.36 (0.62–2.97), p = 0.438	Small-cohort sensitivity; imprecise and non-significant
GSE328930 HOT-ICI	ICI pathologic non-response	cd8_tls_only	28	13	0.513	42.6	CD8/TLS immune access: OR 1.06 (0.50–2.27), p = 0.871	Small-cohort sensitivity; imprecise and non-significant
GSE328930 HOT-ICI	ICI pathologic non-response	core_plus_cd8_tls	28	13	0.600	43.9	barrier core: OR 1.54 (0.57-4.17), p=0.394; CD8/TLS immune access: OR 0.82 (0.31-2.17), p=0.688	Small-cohort sensitivity; imprecise and non-significant
GSE328930 HOT-ICI	ICI pathologic non-response	barrier_exclusion_score	28	13	0.646	41.6	barrier-exclusion score: OR 1.52 (0.68–3.39), p = 0.307	Small-cohort sensitivity; imprecise and non-significant
IMvigor210	anti-PD-L1 non-response	incremental test: CD8/TLS only vs core + CD8/TLS	298		0.604 to 0.627	delta −2.3	LRT p = 0.038; delta AUC = 0.024	Nested-model comparison; exploratory
GSE176307	real-world ICI non-response	incremental test: CD8/TLS only vs core + CD8/TLS	88		0.582 to 0.684	delta −2.1	LRT p = 0.042; delta AUC = 0.102	Nested-model comparison; exploratory

Abbreviations are defined in the manuscript and S1 Table.

**Table 6:** Meta-analysis and revision sensitivity audit.

Analysis	Cohorts/comparisons	N/events	Estimate	Heterogeneity	Verification file	Interpretation
Barrier-core fixed effect	IMvigor210; GSE176307; GSE328930 HOT-ICI	414/315	OR 1.14 (95% CI 0.91–1.44), p = 0.246	I2 = 0.0%	S1 File/analysis/clinical/original_barrier_exclusion_models.csv	Pure barrier core was not significant
Barrier-exclusion fixed effect	IMvigor210; GSE176307; GSE328930 HOT-ICI	414/315	OR 1.52 (95% CI 1.20–1.92), p < 0.001	I2 = 0.0%; tau2 = 0.000000	S1 File/analysis/meta/barrier_exclusion_fixed_and_random_meta_sensitivity.csv	Exploratory pooled support; low I2 is not proof of homogeneity with only two or three cohorts
Barrier-exclusion random effects (REML)	IMvigor210; GSE176307; GSE328930 HOT-ICI	414/315	OR 1.52 (95% CI 1.20–1.92), p < 0.001	I2 = 0.0%; tau2 = 0.000006	S1 File/analysis/meta/barrier_exclusion_fixed_and_random_meta_sensitivity.csv	Exploratory pooled support; low I2 is not proof of homogeneity with only two or three cohorts
Barrier-exclusion fixed effect excluding GSE328930	IMvigor210; GSE176307	386/302	OR 1.52 (95% CI 1.19–1.94), p < 0.001	I2 = 0.0%; tau2 = 0.000000	S1 File/analysis/meta/barrier_exclusion_fixed_and_random_meta_sensitivity.csv	Exploratory pooled support; low I2 is not proof of homogeneity with only two or three cohorts
Barrier-exclusion random effects excluding GSE328930	IMvigor210; GSE176307	386/302	OR 1.52 (95% CI 1.19–1.94), p < 0.001	I2 = 0.0%; tau2 = 0.000006	S1 File/analysis/meta/barrier_exclusion_fixed_and_random_meta_sensitivity.csv	Exploratory pooled support; low I2 is not proof of homogeneity with only two or three cohorts
Weight-grid robustness	12 prespecified sensitivity combinations	3 cohorts per combination	12/12 pooled directions positive and p < 0.05	Not used to select coefficients	S1 File/analysis/meta/barrier_exclusion_weight_grid_meta_models.csv	Supports robustness only; coefficients 0.50 and 0.25 were not optimized
Coefficient prespecification audit	Spatial-context definition versus all response cohorts	Not applicable	CD8 weight = 0.50; TLS/B-cell weight = 0.25	Fixed before response-cohort analysis	S1 File/analysis/meta/meta_and_weight_sensitivity_report.md	Coefficients were carried forward unchanged and never tuned or refit
Patient/sample identifier overlap audit	Six pairwise comparisons among TCGA-BLCA, IMvigor210, GSE176307 and GSE328930	6/6 pairs	No duplicate normalized public identifiers detected	Direct identity linkage unavailable across independently anonymized studies	S1 File/analysis/overlap/clinical_cohort_pairwise_identifier_overlap.csv	No evidence of overlap in public metadata; not a proof based on identifiable patient linkage

Abbreviations are defined in the manuscript and S1 Table. Verification paths identify the corresponding archived output within S1 File.

### Sensitivity analyses define the boundary of the translation score

In IMvigor210, adding the barrier core to a CD8/TLS-only model changed AUC from 0.604 to 0.627 (likelihood-ratio p = 0.038), and the barrier-exclusion score achieved AUC = 0.632. In GSE176307, AUC changed from 0.582 to 0.684 for core-plus-CD8/TLS (likelihood-ratio p = 0.042), while the barrier-exclusion score achieved AUC = 0.685. These are exploratory within-cohort comparisons, not externally calibrated prediction models. In the smaller GSE328930 HOT-ICI subset, the barrier-exclusion AUC was 0.646, but the wide confidence interval and small sample preclude strong inference (Table 5).

**Fig 7:**
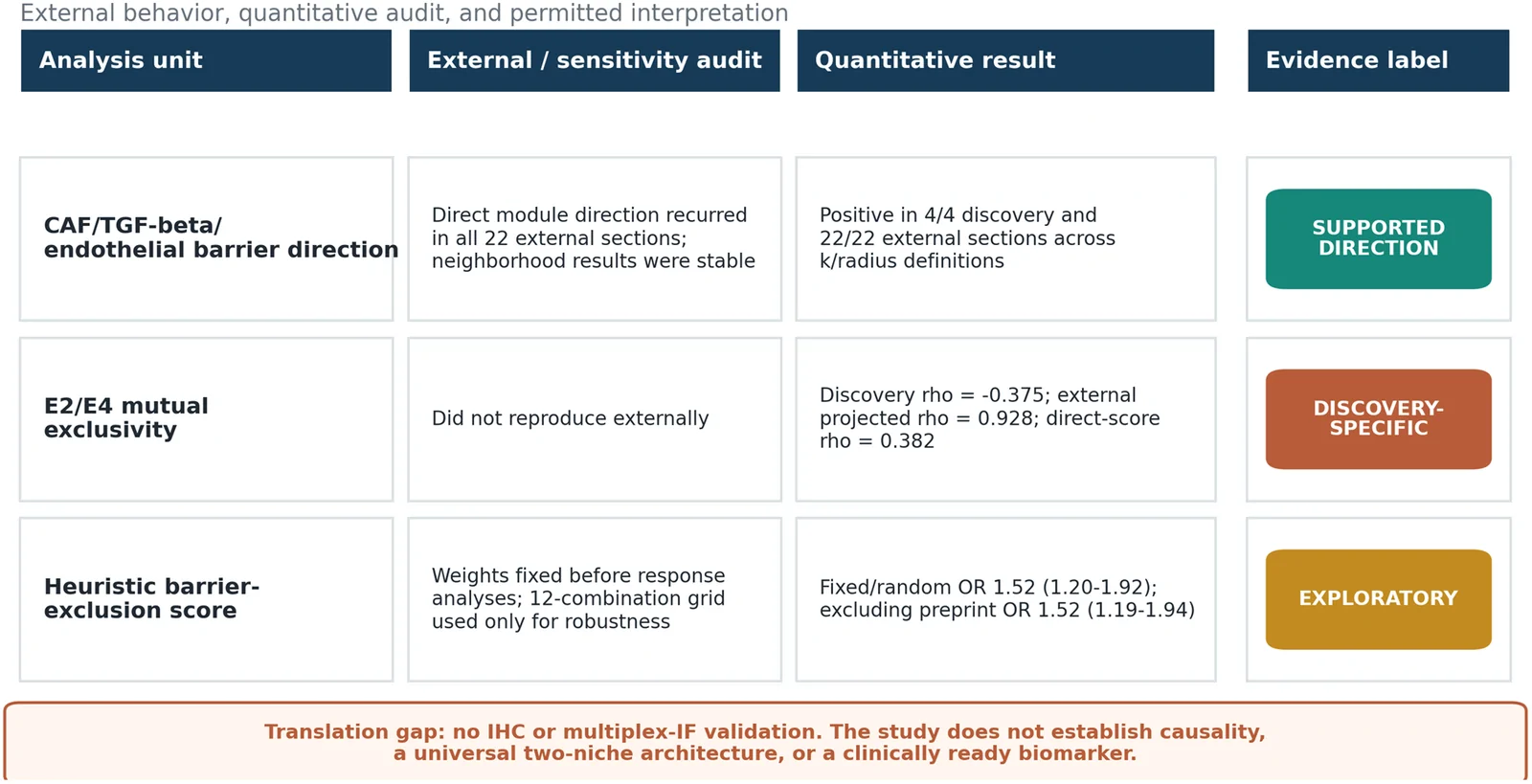
Evidence-strength summary. The figure distinguishes the recurrent spatial barrier direction, the cohort-dependent hypoxic epithelial relationship and the heuristic barrier-exclusion translation score. No causal, universal two-niche or clinically validated biomarker claim is made.

The coefficients 0.50 for CD8 cytotoxicity and 0.25 for TLS/B-cell activity were fixed before any response-cohort analysis and were never tuned or refit. Across a 12-combination coefficient grid, all pooled estimates remained directionally positive with p < 0.05. This grid is a robustness analysis, not an optimization procedure. Spatially, raw CD8/TLS depletion was not universal, particularly in GSE319536. The evidence therefore supports a recurrent barrier direction and a separate, hypothesis-generating immune-resistance translation score, not a universal immune-excluded spatial state (Fig 7; S3 Fig; Table 6).

## Discussion

This public multi-cohort study identifies a recurrent CAF/TGF-beta/endothelial barrier direction associated with immune exclusion and checkpoint resistance in bladder cancer. The main conclusion is narrower than the original wording. The four-section discovery NMF separated barrier-enriched E2 from hypoxic epithelial E4, but dominant component assignment was strongly associated with section identity and the E2/E4 spatial separation did not reproduce externally. GSE319536 instead supported recurrence of the direct barrier direction across 22 sections. The robust unit is therefore the CAF/TGF-beta/endothelial direction, not a universal pair of mutually exclusive niches.

The external contradiction is biologically informative. In GSE171351, E2 and E4 were negatively correlated and almost completely disjoint, whereas in GSE319536 the projected components were strongly positively correlated and the direct barrier and hypoxic epithelial scores also co-varied. Differences in tissue composition, section sampling, disease state, spatial scale, preprocessing and cohort-level lineage architecture could all alter whether stromal and hypoxic epithelial programs separate or co-occur. We therefore avoid the term companion niche as a general claim and treat the E2/E4 relationship as cohort dependent.

Neighborhood sensitivity strengthens the narrower barrier result. The CAF/ECM, TGF-beta/EMT and endothelial directions were preserved with k = 4, 6, 8 and 12 and with a radius-based graph in both spatial cohorts. This robustness does not make Euclidean proximity equivalent to histologic adjacency, and it does not demonstrate a physical barrier without pathology annotation. It does show that the reported module direction is not an artifact of the prespecified k = 6 choice.

The present work also differs from, and should be interpreted alongside, recent spatial studies. Recent work has linked spatial relationships within the tumor microenvironment to response to immune checkpoint inhibition, reviewed the potential predictive value of spatial biomarkers, and identified profibrotic ecotypes associated with tumor immunity across cancer types [26–28]. Spatial EcoTyper learns recurrent multicellular states from cell-type-specific expression covariation across large collections [19], whereas our discovery analysis factorizes locked module and neighborhood scores from four GSE171351 sections. GSE319536 was used here as an external test of a predefined barrier direction; the original atlas addresses broader lineage-associated tumor states and immune architecture across 22 tumors [20]. Our narrower contribution is the transparent projection of a predefined barrier direction into public clinical cohorts, accompanied by explicit negative replication and sensitivity analyses. No Spatial EcoTyper-derived score was recovered or benchmarked in the present study.

The clinical results require similar restraint. In IMvigor210, E2-high was associated with non-response in the univariable analysis, but the fully adjusted p value was 0.051 and the delta AUC was only 0.009. This is suggestive, not conclusive. In TCGA-BLCA, continuous E2 was associated with survival, but the adjusted top-quartile contrast was not. Dichotomization can reduce information and power, and the data do not support a clinically meaningful prognostic threshold.

The barrier-exclusion score provides a more consistent clinical bridge but remains heuristic. Its 0.50 and 0.25 coefficients were inherited from the spatial-context definition before response analyses and were never tuned in the clinical cohorts. The fixed- and random-effects summaries and the analysis excluding GSE328930 were stable, but only two or three heterogeneous cohorts contributed. GSE176307 is a real-world metastatic cohort, IMvigor210 is a metastatic atezolizumab cohort and GSE328930 is a small neoadjuvant cohort. Consequently, low I2 has little power to exclude heterogeneity, and the pooled OR should not be interpreted as validation of a universal predictive biomarker.

Strengths of the study include locked module definitions, explicit gene and coverage reporting, separation of spatial and clinical evidence, sample-aware analyses, alternative-neighborhood checks, rank/initialization diagnostics, fixed- and random-effects sensitivity analyses, and preservation of negative results. The revised tables separate cohort-specific model comparisons from pooled estimates and remove datasets and variables that were not part of the reported analyses.

The limitations are substantial. The discovery NMF is based on four sections and is strongly associated with section identity. Neither spatial cohort contains prospective ICI outcomes, and bulk projection cannot establish spatial adjacency. Clinical models are retrospective and depend on public annotation quality. The response cohorts differ in stage, treatment and study design, and one is a small neoadjuvant cohort. Raw CD8/TLS depletion was not spatially universal. Most importantly for translation, the study lacks orthogonal pathology validation by immunohistochemistry or multiplex immunofluorescence. Direct colocalization of CAF, TGF-beta pathway, endothelial, epithelial, CD8/GZMB and TLS markers in pretreatment tissue is required before a physical barrier mechanism can be established.

In conclusion, the study supports a recurrent CAF/TGF-beta/endothelial barrier direction associated with immune exclusion and checkpoint resistance in bladder cancer. It does not establish causal underpinnings, a universal two-niche architecture or a clinically ready biomarker. The barrier-exclusion score is a transparent hypothesis-generating translation axis that now requires prospective, pathology-linked validation.

## Supporting information

S1 File Analysis scripts, processed outputs and revision audit files. The rebuilt ZIP archive uses short ASCII paths and standard deflate compression and contains a root manifest, original and revision analysis scripts, processed tables and verification reports.
(ZIP)

S1 Table Gene signatures, pairwise overlap and cohort coverage. The workbook lists exact gene symbols for all 11 modules, module sizes, pairwise overlap, cohort-specific coverage summaries and record-level coverage.
(XLSX)

S1 Fig NMF rank and initialization stability. Ranks 3–7 were compared using 30 initializations per rank. Reconstruction error, consensus cophenetic correlation, PAC, dispersion, matched-component similarity and section association are summarized; rank 5 is marked as the prespecified discovery representation.
(PNG)

S2 Fig Neighborhood-definition sensitivity. Barrier scores and module directions obtained with k = 4, 6, 8 and 12 and a radius graph are compared within GSE171351 and GSE319536. The CAF/ECM, TGF-beta/EMT and endothelial directions were preserved across methods.
(PNG)

S3 Fig Barrier-exclusion weight and meta-analysis sensitivity. Cohort estimates, fixed- and random-effects summaries, the analysis excluding GSE328930 and the 12-combination coefficient grid are shown. The grid assesses robustness and was not used for coefficient optimization.
(PNG)
