# Supplementary material for: A recurrent cancer-associated fibroblast/TGF-beta/endothelial barrier direction is associated with immune exclusion and checkpoint resistance in bladder cancer: a public multi-cohort transcriptomic study
Source: PLoS One. 2026 Sep 22;21(9):e0358043. doi: 10.1371/journal.pone.0358043 (PMC13596827; doi:10.1371/journal.pone.0358043)
Supplement: Supplementary file 1 [file pone.0358043.s007.docx]

Response to Reviewers

**Manuscript ID:** PONE-D-26-26495

*A recurrent cancer-associated fibroblast/TGF-beta/endothelial barrier direction is associated with immune exclusion and checkpoint resistance in bladder cancer: a public multi-cohort transcriptomic study*

# Overview for the Editor

Dear Dr. Shoari,

We thank you and both reviewers for the careful and constructive assessment of our manuscript. The revision narrows the central claim from a universal two-niche mechanism to a recurrent CAF/TGF-beta/endothelial barrier direction and explicitly reports the external non-replication of E2/E4 mutual exclusivity. We added NMF rank/initialization diagnostics, alternative-neighborhood analyses, fixed- and random-effects meta-analysis with exclusion of the preprint cohort, a coefficient-grid robustness audit and a public-identifier overlap audit. We removed unused datasets and the residual nature_SE_score field, separated cohort-specific model comparisons from pooled sensitivity analyses, supplied an auditable gene-signature/coverage workbook, and rebuilt the previously unreadable S1 archive. We also removed funding text from the manuscript, retained the ethics statement only in Methods, softened borderline clinical interpretations and completed a full scientific-English edit. All changes are visible in the marked manuscript, and the locations below refer to the final line-numbered clean manuscript.

# Journal Requirements

JR1 PLOS ONE style and file naming

**Reviewer’s Comment.** Please ensure that the revised manuscript meets PLOS ONE style requirements, including file naming.

**Response.** We have reformatted the clean and marked manuscripts as line-numbered Word files, standardized the title page, headings, tables, supporting-information labels and figure captions, and assigned upload-ready filenames keyed to PONE-D-26-26495. The revised manuscript contains six editable tables and separates the clean and marked versions.

**Changes in Text.** Formatting and naming changes apply throughout the clean and marked manuscripts. The upload manifest identifies the exact files supplied for Manuscript, Revised Manuscript with Track Changes, Response to Reviewers and Supporting Information.

JR2 Funding text removed from the manuscript

**Reviewer’s Comment.** Please remove any funding-related text from the manuscript; funding information will be published from the online Financial Disclosure.

**Response.** We have removed the Funding heading and all funding statements or placeholders from the manuscript. Funding information is retained only in the online submission form.

**Changes in Text.** Deleted throughout the manuscript. Because this requirement concerns deletion, there is no surviving manuscript line to quote. The clean-manuscript audit confirms that neither a Funding section nor funding-disclosure text remains.

JR3 Ethics statement retained only in Methods

**Reviewer’s Comment.** The ethics statement should appear only in the Methods section.

**Response.** We have retained a single public-data ethics statement under ‘Software, reproducibility and ethics’ and removed the duplicate statement outside Methods.

**Changes in Text.** Clean manuscript, p. 11, lines 234-238.

This study used publicly available, de-identified transcriptomic and clinical datasets and did not involve new patient recruitment or intervention. All source datasets were accessed for research use under their original data-access conditions.

JR4 Supporting Information archive rebuilt

**Reviewer’s Comment.** The Supporting Information file S1_File_analysis_scripts_and_key_outputs.zip could not be opened. Please revise and re-upload it.

**Response.** We rebuilt S1 File as a standard DEFLATE ZIP with short ASCII paths, a root README, a machine-readable manifest and SHA-256 checksums. The archive contains 114 members, has no nested archive, contains no references to excluded cohorts or the removed benchmark field, and passed Python CRC validation plus an independent extraction/checksum test.

**Changes in Text.** Clean manuscript, p. 31, lines 539-541. Rebuilt file: S1_File_reproducibility_scripts_and_key_outputs.zip; root audit files: README.txt, MANIFEST.csv and SHA256SUMS.txt.

S1 File. Analysis scripts, processed outputs and revision audit files. The rebuilt ZIP archive uses short ASCII paths and standard deflate compression and contains a root manifest, original and revision analysis scripts, processed tables and verification reports.

# Responses to Reviewer 1

R1.1 External E2/E4 non-replication

**Reviewer’s Comment.** GSE319536 does not reproduce the discovery-cohort E2/E4 separation. Projected E2 and E4 are positively correlated, so the ‘companion niche’ interpretation requires substantial qualification.

**Response.** We agree and have changed the central interpretation. E2/E4 mutual exclusivity is now described as a discovery-cohort decomposition rather than a universal two-niche architecture. In GSE319536, projected E2 and E4 were positively correlated (Spearman rho=0.928), and the direct barrier and hypoxic epithelial scores also co-varied. The externally recurrent unit is therefore the direct CAF/TGF-beta/endothelial barrier direction, which was preserved in 22 of 22 sections, not E2/E4 separation.

**Changes in Text.** Title and Abstract, pp. 1-2, lines 3-31; Results, pp. 13-14, lines 272-294; Discussion, pp. 17-18, lines 354-371; Table 3, p. 27, line 527.

The external cohort reproduced the direct CAF/TGF-beta/endothelial barrier direction in 22 of 22 sections but did not reproduce E2/E4 mutual exclusivity: projected E2 and E4 were positively correlated (rho=0.928).

R1.2 Heuristic score weights

**Reviewer’s Comment.** The barrier-exclusion score weights 0.50 and 0.25 appear arbitrary and require justification or optimization.

**Response.** We now state explicitly that the weights are transparent heuristics, not data-driven coefficients. They were inherited unchanged from the spatial-context definition, fixed before any response-cohort analysis, and never tuned or refit. We added a 12-combination weight grid solely as a robustness check; all pooled directions were positive with p<0.05, but the grid was not used to choose the reported coefficients.

**Changes in Text.** Methods, p. 8, lines 160-164; Results, p. 17, lines 347-352; Discussion, p. 18, lines 389-397; Table 6, p. 30, line 536; Fig S3.

The coefficients 0.50 and 0.25 are heuristic rather than data-driven. They were carried forward unchanged from the spatial-context exclusion definition, fixed before any response-cohort analysis, and never tuned, optimized or refit.

R1.3 IMvigor210 result characterized as suggestive

**Reviewer’s Comment.** The fully adjusted IMvigor210 result is borderline (p=0.051; delta AUC=0.009) and should not be described confidently.

**Response.** We agree. The title, Abstract, Results, Discussion and Fig 4 caption now describe the fully adjusted E2-high result as suggestive but not statistically conclusive. We report the OR, confidence interval, p value and delta AUC together and do not present E2-high as an independently validated biomarker.

**Changes in Text.** Abstract, p. 2, lines 26-28; Results, p. 15, lines 297-306; Discussion, p. 18, lines 383-387; Table 4, p. 28, line 530; Fig 4 caption, p. 32, lines 559-562.

In IMvigor210, top-quartile E2-high tumors showed suggestive, but not statistically conclusive, evidence of anti-PD-L1 non-response after full biologic covariate adjustment (OR=2.57, 95% CI 1.00-6.64, p=0.051; delta AUC=0.009).

R1.4 Heterogeneous-cohort meta-analysis

**Reviewer’s Comment.** The meta-analysis mixes metastatic, real-world and neoadjuvant cohorts. Please include a sensitivity analysis excluding the preprint cohort and clearly label pooled results exploratory.

**Response.** We recalculated both inverse-variance fixed-effect and REML random-effects models and added fixed- and random-effects analyses excluding GSE328930. The three-cohort estimates were OR=1.52 (95% CI 1.20-1.92), and exclusion of the preprint cohort yielded OR=1.52 (95% CI 1.19-1.94). We now label every pooled estimate exploratory and note that low I2 from only two or three cohorts does not establish homogeneity.

**Changes in Text.** Methods, p. 10, lines 207-218; Results, p. 16, lines 334-342; Discussion, p. 18, lines 389-397; Table 6, p. 30, line 536; Fig S3.

The exploratory three-cohort fixed-effect and REML random-effects summaries were nearly identical (OR=1.52, 95% CI 1.20-1.92; I2=0%; tau-squared approximately 0). Excluding the preprint cohort yielded OR=1.52 (95% CI 1.19-1.94).

R1.5 Absence of orthogonal spatial validation

**Reviewer’s Comment.** The lack of orthogonal spatial validation by IHC or multiplex IF is a major gap for a spatial hypothesis.

**Response.** We agree and now identify this as the principal translational limitation. The revised manuscript states that Euclidean transcriptomic proximity is not equivalent to histologic adjacency and that a physical barrier mechanism cannot be established without pathology-level colocalization.

**Changes in Text.** Discussion, p. 19, lines 403-412.

Most importantly for translation, the study lacks orthogonal pathology validation by immunohistochemistry or multiplex immunofluorescence. Direct colocalization of CAF, TGF-beta pathway, endothelial, epithelial, CD8/GZMB and TLS markers in pretreatment tissue is required before a physical barrier mechanism can be established.

R1.6 Exact gene signatures supplied

**Reviewer’s Comment.** The gene signatures are not listed. Please provide a supplementary table with the full gene sets for all modules.

**Response.** We created Supplementary Table S1 as a six-sheet workbook containing exact gene symbols for all 11 modules, module sizes, sources, pairwise overlap, cohort-level coverage summaries and record-level coverage. The definitions contain 117 unique genes; CAF/ECM and TGF-beta/EMT share only COL1A1 and COL3A1.

**Changes in Text.** Methods, p. 6, lines 111-118; Results, p. 12, lines 250-256; Supporting information, p. 31, lines 542-544; Supplementary Table S1.

Exact gene symbols, module sizes, pairwise overlaps and cohort-specific gene coverage are reported in Supplementary Table S1. The 11 modules contained 117 unique genes; CAF/ECM and TGF-beta/EMT shared COL1A1 and COL3A1.

R1.7 Neighborhood-definition robustness

**Reviewer’s Comment.** The nearest-neighbor definition (k=6, Euclidean) is arbitrary; please justify it or test alternatives.

**Response.** We retained k=6 as the prespecified local summary because it approximates the immediate Visium lattice neighborhood while remaining applicable to incomplete capture geometry. We repeated the analysis with k=4, 8 and 12 and a radius graph defined by each section’s median sixth-neighbor distance. Median agreement with k=6 ranged from 0.972 to 0.985 in GSE171351 and from 0.981 to 0.993 in GSE319536; the barrier directions remained positive in 4/4 and 22/22 sections for every definition.

**Changes in Text.** Methods, p. 7, lines 130-136; Results, p. 14, lines 286-294; Discussion, p. 18, lines 368-373; Table 3, p. 27, line 527; Fig S2.

For k=4, 6, 8 and 12 and a radius graph defined from the median sixth-neighbor distance, CAF/ECM, TGF-beta/EMT and endothelial associations were positive in 4 of 4 GSE171351 sections and 22 of 22 GSE319536 sections for every method.

R1.8 Continuous versus top-quartile TCGA results

**Reviewer’s Comment.** Continuous E2 is significant in TCGA-BLCA, but top-quartile E2 is not significant after adjustment. Please explain this discrepancy.

**Response.** We now present the continuous and thresholded estimates side by side and interpret the discrepancy as loss of information and power from dichotomization rather than evidence for a validated threshold. The revised manuscript explicitly states that the data do not support a clinically meaningful prognostic cutoff.

**Changes in Text.** Results, p. 15, lines 308-316; Discussion, p. 18, lines 383-388; Table 4, p. 28, line 530; Fig 5 caption, p. 32, lines 563-566.

In TCGA-BLCA, continuous E2 was associated with survival, but the adjusted top-quartile contrast was not. Dichotomization can reduce information and power, and the data do not support a clinically meaningful prognostic threshold.

R1.9 Dense clinical presentation simplified

**Reviewer’s Comment.** Figures 4-7 are overly dense, and redundant covariate tables should be reduced to a single summary.

**Response.** We simplified the main clinical presentation by retaining decision-relevant estimates, rewriting the figure captions around evidence strength, and separating the cohort-specific model comparisons from pooled and sensitivity results. Detailed NMF, neighborhood and weight/meta diagnostics are now in Figs S1-S3 rather than being added to the main clinical panels. The revised tables remove the former combined layout and redundant covariate blocks.

**Changes in Text.** Results, pp. 15-17, lines 297-352; Table 4, p. 28; Table 5, p. 29; Table 6, p. 30; figure captions, pp. 32-33, lines 559-585.

# Responses to Reviewer 2

R2.1 NMF rank and initialization stability

**Reviewer’s Comment.** The discovery ecotype is derived from only four sections and may be dominated by sample identity. Please provide NMF stability and rank-sensitivity diagnostics.

**Response.** We added ranks 3-7 with 30 initializations per rank and report reconstruction error, consensus cophenetic correlation, PAC, dispersion and matched-component similarity. For rank 5, cophenetic correlation was 0.990, PAC 0.123, dispersion 0.893 and median matched-component cosine similarity 0.960. We also report dominant-component versus section identity (Cramer’s V=0.944) and now retain rank 5 only as the prespecified discovery representation, not as uniquely optimal.

**Changes in Text.** Methods, p. 7, lines 137-147; Results, p. 13, lines 261-269; Discussion, pp. 17 and 19, lines 354-361 and 403-404; Table 3, p. 27; Fig S1.

Rank 5 was retained as the submitted discovery representation rather than as a uniquely optimal solution. Dominant rank-5 component assignment remained strongly associated with section identity (Cramer’s V=0.944), demonstrating that the four-section design substantially influenced the NMF architecture.

R2.2 Universal two-niche claim withdrawn

**Reviewer’s Comment.** The central two-niche claim does not replicate externally and should not remain the main framing.

**Response.** We agree. We removed the universal two-niche framing from the title, Abstract and conclusions. The revised title refers to a recurrent barrier direction; E2/E4 mutual exclusivity is explicitly limited to GSE171351, while GSE319536 is presented as negative replication for E2/E4 separation and positive recurrence for the direct barrier direction.

**Changes in Text.** Title and Abstract, pp. 1-2, lines 3-31; Results, pp. 13-14; Discussion, pp. 17-20, lines 354-414; Table 3, p. 27.

These results identify a recurrent barrier direction rather than a universal two-niche architecture.

R2.3 Weight prespecification confirmed

**Reviewer’s Comment.** Please state whether the 0.50 and 0.25 coefficients were fixed before any response-cohort analysis and whether they were tuned or refit.

**Response.** We traced the score provenance and confirm that both coefficients were carried forward unchanged from the spatial-context definition before IMvigor210, GSE176307 or GSE328930 response modeling. They were never tuned or refit. The coefficient-grid analysis was performed only after review as a robustness audit and did not alter the score.

**Changes in Text.** Methods, p. 8, lines 160-164; Results, p. 17, lines 347-352; Table 6, p. 30; S1 File/analysis/meta/meta_and_weight_sensitivity_report.md.

They were carried forward unchanged from the spatial-context exclusion definition, fixed before any response-cohort analysis, and never tuned, optimized or refit in IMvigor210, GSE176307 or GSE328930.

R2.4 Borderline findings and meta-analysis recalculated

**Reviewer’s Comment.** Several borderline findings are stated too confidently; the HOT-ICI cohort is small, the pooled ORs require verification, and a random-effects analysis is needed.

**Response.** We revised all borderline language, identified the 28-patient HOT-ICI analysis as imprecise and non-significant, and independently recalculated the pooled effects. The fixed- and random-effects estimates are nearly identical because the fitted between-study variance is approximately zero, but we explicitly caution that heterogeneity cannot be excluded with only two or three cohorts. We also added fixed- and random-effects models excluding the preprint cohort.

**Changes in Text.** Abstract, p. 2; Results, pp. 15-17; Discussion, pp. 18-19; Tables 5-6, pp. 29-30; Fig S3.

R2.5 Relationship to Spatial EcoTyper and the GSE319536 atlas

**Reviewer’s Comment.** Please clarify the relationship to recent Spatial EcoTyper work and the Yu et al. GSE319536 atlas, especially because datasets overlap.

**Response.** We added a direct methodological comparison. Spatial EcoTyper learns cell-type-specific covariation across large multisample collections, whereas our NMF used locked module and neighborhood features from four GSE171351 sections. GSE319536 is used here only as an external test of a predefined direct barrier direction and projected E2/E4 components. We removed the residual nature_SE_score field and now state explicitly that no Spatial EcoTyper-derived score was recovered or benchmarked.

**Changes in Text.** Introduction, p. 3, lines 61-70; Discussion, p. 18, lines 374-382; revised Table 2, p. 26.

Our narrower contribution is the transparent projection of a predefined barrier direction into public clinical cohorts, accompanied by explicit negative replication and sensitivity analyses. No Spatial EcoTyper-derived score was recovered or benchmarked in the present study.

R2.6 Unused datasets deleted

**Reviewer’s Comment.** Table 2 lists GSE326226, GSE299573, GSE305629, GSE326225 and GSE135337 even though they are not analyzed elsewhere.

**Response.** We removed all five unused accessions from the manuscript, Table 2 and supporting audit package. The revised Methods and Tables 1-2 contain only the six datasets actually analyzed: GSE171351, GSE319536, TCGA-BLCA, IMvigor210, GSE176307 and GSE328930/DUTRENEO.

**Changes in Text.** Methods, p. 5, lines 82-93; Tables 1-2, pp. 25-26. A repository-wide residual scan found none of the five deleted accessions in the revised deliverables.

No other datasets were included in the reported analyses (Tables 1 and 2).

R2.7 Causal wording removed

**Reviewer’s Comment.** The term ‘underlies’ in the title and Abstract implies a causal or mechanistic conclusion not supported by this retrospective computational design.

**Response.** We agree and replaced causal wording with associational and recurrence language throughout. The title now uses ‘is associated with’; the conclusions state explicitly that the study does not establish causal underpinnings, a universal two-niche architecture or a clinically ready biomarker.

**Changes in Text.** Title, p. 1, lines 3-5; Abstract, pp. 1-2; Discussion and conclusion, pp. 17-20, lines 354-414; figure captions, pp. 31-33.

It does not establish causal underpinnings, a universal two-niche architecture or a clinically ready biomarker.

R2.8 Signature visibility, overlap and coverage

**Reviewer’s Comment.** Please provide exact gene symbols for every module, module sizes, cohort coverage and overlap, particularly CAF/ECM versus TGF-beta/EMT.

**Response.** We supplied the requested information in Supplementary Table S1. The workbook includes exact symbols, module-level summary, pairwise overlap, six-cohort coverage summary and record-level coverage. CAF/ECM and TGF-beta/EMT share COL1A1 and COL3A1; other module pairs do not overlap.

**Changes in Text.** Methods, p. 6, lines 111-118; Results, p. 12, lines 250-256; Supplementary Table S1.

R2.9 Patient/sample overlap audit

**Reviewer’s Comment.** Please confirm whether TCGA-BLCA, IMvigor210, GSE176307 and GSE328930 contain any patient-level overlap.

**Response.** We performed six pairwise identifier comparisons after normalization. No duplicate public sample or patient identifiers were detected. Because each study used independently anonymized identifiers, we cannot prove identity-level non-overlap; we therefore report this as no evidence of overlap in public metadata rather than a definitive linkage result.

**Changes in Text.** Methods, p. 9, lines 191-194; Results, p. 12, lines 255-258; Table 6, p. 30; S1 File/analysis/overlap/clinical_cohort_pairwise_identifier_overlap.csv.

No duplicate normalized public sample or patient identifiers were detected across the six pairwise cohort comparisons. Direct cross-study identity linkage was not possible because the public identifiers were independently anonymized.

R2.10 Table 5 split into cohort and pooled analyses

**Reviewer’s Comment.** Table 5 combines cohort-specific model comparisons and meta-analysis results; please separate them for readability.

**Response.** We split the original table into two independently interpretable tables. Revised Table 5 contains only single-cohort response-model comparisons and incremental tests. New Table 6 contains the fixed/random meta-analysis, exclusion of the preprint cohort, weight-grid robustness, coefficient-prespecification audit and patient/sample-overlap audit.

**Changes in Text.** Table 5, p. 29, lines 533-534; Table 6, p. 30, lines 536-537; Results, p. 16, lines 334-352.

The revised tables separate cohort-specific model comparisons from pooled estimates and remove datasets and variables that were not part of the reported analyses.

R2.11 Scientific English and readability

**Reviewer’s Comment.** The manuscript requires thorough professional English-language editing and simplification of awkward or overly complex sentences.

**Response.** We performed a full scientific-English and terminology pass after completing the statistical revisions. We shortened complex sentences, standardized cohort and score names, removed causal overstatement, and separated spatial-mechanistic evidence from exploratory clinical translation. The clean and marked manuscripts were then rendered page by page and reviewed for text overflow, table clipping and inconsistent formatting.

**Changes in Text.** Throughout the manuscript; representative revised framing appears in the Abstract (pp. 1-2, lines 16-31) and Discussion (pp. 17-20, lines 354-414).

# Modification and Execution Checklist

- All four journal requirements addressed and mapped to deliverables.
- All Reviewer 1 comments R1.1-R1.9 answered.
- All Reviewer 2 comments R2.1-R2.11 answered.
- Unused accessions and nature_SE_score absent from revised manuscript, tables and S1 archive.
- Table 5 contains only single-cohort comparisons; Table 6 contains meta-analysis and revision sensitivity audits.
- NMF stability, neighborhood robustness, external non-replication and patient-overlap audits have verification paths in Tables 3 and 6.
- Clean and marked manuscripts rendered without clipped text or table overflow.
- S1 archive passed CRC, checksum and independent extraction tests.
- Supplementary Table S1 lists exact genes, overlap and coverage.

**Sincerely,**
Yuye Wu, on behalf of all authors
