# Supplementary material for: A recurrent cancer-associated fibroblast/TGF-beta/endothelial barrier direction is associated with immune exclusion and checkpoint resistance in bladder cancer: a public multi-cohort transcriptomic study
Source: PLoS One. 2026 Sep 22;21(9):e0358043. doi: 10.1371/journal.pone.0358043 (PMC13596827; doi:10.1371/journal.pone.0358043)
Supplement: Peer review history

# Peer review: Decision Letter 0

**DOI:** 10.1371/journal.pone.0358043.r001

**Authors:** Alireza Shoari

17 Aug 2026

Dear Dr. Yuye,

Thank you for submitting your manuscript to PLOS ONE. After careful consideration, we feel that it has merit but does not fully meet PLOS ONE’s publication criteria as it currently stands. Therefore, we invite you to submit a revised version of the manuscript that addresses the points raised during the review process.

Please submit your revised manuscript by Oct 16 2026 11:59PM. If you will need more time than this to complete your revisions, please reply to this message or contact the journal office at plosone@plos.org. When you're ready to submit your revision, log on to https://www.editorialmanager.com/pone/ and select the 'Submissions Needing Revision' folder to locate your manuscript file.

- A letter that responds to each point raised by the academic editor and reviewer(s). You should upload this letter as a separate file labeled 'Response to Reviewers'.
- A marked-up copy of your manuscript that highlights changes made to the original version. You should upload this as a separate file labeled 'Revised Manuscript with Track Changes'.
- An unmarked version of your revised paper without tracked changes. You should upload this as a separate file labeled 'Manuscript'.

If you would like to make changes to your financial disclosure, please include your updated statement in your cover letter. Guidelines for resubmitting your figure files are available below the reviewer comments at the end of this letter.

If applicable, we recommend that you deposit your laboratory protocols in protocols.io to enhance the reproducibility of your results. Protocols.io assigns your protocol its own identifier (DOI) so that it can be cited independently in the future. For instructions see: https://journals.plos.org/plosone/s/submission-guidelines#loc-laboratory-protocols. Additionally, PLOS ONE offers an option for publishing peer-reviewed Lab Protocol articles, which describe protocols hosted on protocols.io. Read more information on sharing protocols at https://plos.org/protocols?utm_medium=editorial-email&utm_source=authorletters&utm_campaign=protocols.

As the corresponding author, your ORCID iD is verified in the submission system and will appear in the published article. PLOS supports the use of ORCID, and we encourage all coauthors to register for an ORCID iD and use it as well. Please encourage your coauthors to verify their ORCID iD within the submission system before final acceptance, as unverified ORCID iDs will not appear in the published article. *Only* the individual author can complete the verification step; PLOS staff *cannot* verify ORCID iDs on behalf of authors.

We look forward to receiving your revised manuscript.

Kind regards,

Alireza Shoari

Academic Editor

PLOS One

Journal Requirements:

When submitting your revision, we need you to address these additional requirements.

1. Please ensure that your manuscript meets PLOS ONE's style requirements, including those for file naming. The PLOS ONE style templates can be found at https://journals.plos.org/plosone/s/file?id=wjVg/PLOSOne_formatting_sample_main_body.pdf and https://journals.plos.org/plosone/s/file?id=ba62/PLOSOne_formatting_sample_title_authors_affiliations.pdf

2. Please remove any funding-related text from the manuscript. Please note that funding information should not appear in any section or other areas of your manuscript. We will publish the funding statement that is present in the Financial Disclosure section of the online submission form.

3. Your ethics statement should only appear in the Methods section of your manuscript. If your ethics statement is written in any section besides the Methods, please move it to the Methods section and delete it from any other section. Please ensure that your ethics statement is included in your manuscript, as the ethics statement entered into the online submission form will not be published alongside your manuscript.

4. We are unable to open your Supporting Information file [S1_File_analysis_scripts_and_key_outputs.zip]. Please kindly revise as necessary and re-upload.

If the reviewer comments include a recommendation to cite specific previously published works, please review and evaluate these publications to determine whether they are relevant and should be cited. There is no requirement to cite these works unless the editor has indicated otherwise.

[Note: HTML markup is below. Please do not edit.]

Reviewers' comments:

Reviewer's Responses to Questions

**Comments to the Author**

1. Is the manuscript technically sound, and do the data support the conclusions?

Reviewer #1: Yes

Reviewer #2: Partly

**********

2. Has the statistical analysis been performed appropriately and rigorously?

Reviewer #1: Yes

Reviewer #2: I Don't Know

**********

3. Have the authors made all data underlying the findings in their manuscript fully available?

The PLOS Data policy

Reviewer #1: Yes

Reviewer #2: No

**********

4. Is the manuscript presented in an intelligible fashion and written in standard English?

Reviewer #1: Yes

Reviewer #2: No

**********

Reviewer #1: A transparent, well‑executed re‑analysis of public spatial/bulk data, proposing a CAF/TGF‑β/endothelial barrier niche and a “barrier‑exclusion” score. The two‑layer framework (mechanism vs. translation) is sensible, and negative findings are honestly reported. However, novelty is incremental, and several methodological/interpretational issues require revision.

1. External spatial cohort (GSE319536) does not reproduce E2/E4 separation – E2 and E4 are positively correlated (rho=0.928). The “companion niche” interpretation is weak; discuss why the relationship varies.

2. Barrier‑exclusion score weights (0.5 and 0.25) are arbitrary – no justification or optimisation. State explicitly that these are heuristic, not data‑driven.

3. IMvigor210 E2‑high effect is borderline after full adjustment (p=0.051, ΔAUC=0.009) – rephrase as “suggestive” rather than “associated.”

4. Meta‑analysis mixes heterogeneous cohorts (metastatic, real‑world, neoadjuvant) – include a sensitivity analysis excluding the preprint cohort (GSE328930) and clearly label as exploratory.

5. No orthogonal spatial validation (IHC/IF) – a major gap for a spatial hypothesis. State this as a key limitation for translation.

6. Gene signatures are not listed – provide a supplementary table with full gene sets for all modules.

7. Nearest‑neighbour definition (k=6, Euclidean) is arbitrary – justify or test alternative (radius/graph) approaches.

8. TCGA‑BLCA: continuous E2 significant but top‑quartile not after adjustment – explain why threshold loses significance (non‑linearity? low power?).

9. Overly dense presentation – simplify Figures 4–7 and reduce redundant covariate tables to a single summary.

Reviewer #2: The overall framing of the manuscript, particularly the distinction between the mechanistic layer and the hypothesis-generating clinical layer, is sound, and the manuscript is commendably transparent regarding its negative results. However, several important issues concerning the size and behavior of the discovery cohort, replication of the central spatial finding, statistical interpretation of borderline results, and potential overlap with recently published work based on the same discovery dataset should be addressed before the manuscript can be considered suitable for publication.

1. The spatial ecotype appears to be derived entirely from four tissue sections, and the manuscript's own diagnostics suggest that this limited sample size may substantially influence the results. Specifically, GSE171351 contributes only four Visium sections (4,086 spots) to the rank-5 NMF discovery analysis, while the authors report a strong association between global E2/E4 high-state assignment and sample identity (Cramer's V = 0.660). With only four samples, an NMF solution can potentially be dominated by sample-level effects, including batch effects, tissue quality, or section orientation, rather than by a generalizable biological program. The sample-adjusted within-sample analysis represents a reasonable attempt to mitigate this concern, but it does not fully resolve the issue. In particular, the observation that three of four samples show the expected direction for several features indicates that one sample already deviates from the proposed pattern at the very small sample level. Please therefore (a) report the stability of the NMF components across the 30 initializations, for example using an appropriate consensus or cophenetic stability measure rather than relying only on the lowest Frobenius error from a single solution, and (b) provide a clear justification for selecting rank = 5, including whether alternative ranks were evaluated and how the selected rank compared with those alternatives.

2. The central “two-niche” claim, specifically the mutual exclusivity of E2 and E4, does not appear to replicate in the external cohort, and this issue warrants more than a minor caveat. In GSE171351, E2 and E4 are negatively correlated (Spearman rho = −0.375) and are almost completely spatially disjoint (1,019/1,019/3 spots). In contrast, in GSE319536, the same two axes are positively correlated (rho = 0.928 for the projected components; 0.382 using the direct scores). Thus, the external cohort does not simply show weaker replication; rather, the direction of the association appears to reverse. The current wording, “universal E2/E4 separation was not reproduced,” therefore seems to understate the extent of this discrepancy. At the same time, the replication of the individual CAF/ECM–TGFβ/EMT–endothelial module direction across all 22 samples represents a meaningful and potentially valuable finding. However, the broader interpretation that the “barrier niche is spatially and biologically distinct from a hypoxic companion niche,” which informs a substantial part of the Discussion, appears to rely on a pattern observed in one small cohort and contradicted in the larger external cohort. Please consider either substantially softening the two-niche framing throughout the manuscript, including the title, abstract, and Discussion, in favor of the more defensible concept of a “recurrent barrier direction,” or explicitly presenting the E2/E4 relationship as cohort-dependent and unresolved rather than incorporating it into the model's definitive conclusions.

3. Please explicitly state whether the weights used in the barrier-exclusion score were fixed before any response-cohort analysis was performed. The current formulation, barrier_core − 0.50 × CD8 − 0.25 × TLS, appears to use exactly the same coefficients (0.50 and 0.25) as the earlier spatial-context exclusion score defined in the spatial mechanism analysis, before the ICI-response modeling is introduced. If these weights were carried over unchanged from the spatial formulation and were never tuned or refitted using outcomes from IMvigor210, GSE176307, or GSE328930, this would provide important support for the claim that the score was “locked.” Please state this explicitly, for example by indicating that the 0.50/0.25 weighting was fixed at the spatial-mechanism stage and was not refit using any clinical outcome cohort.

4. Several borderline or non-significant findings appear to be characterized somewhat more confidently than the corresponding statistical estimates warrant. Please consider revising the wording and interpretation of these results. For the IMvigor210 full covariate model, the reported OR is 2.57 with a 95% CI of 1.00–6.64 and p = 0.051. Describing this as “borderline evidence” in the abstract is reasonable; however, the Discussion's characterization as remaining “directionally consistent” could be interpreted as more supportive than warranted by a confidence interval that reaches 1.0. For the GSE328930 HOT-ICI barrier-exclusion analysis, n = 28 with 13 events, the OR is 1.52, 95% CI 0.68–3.39, and p = 0.307, yet this point estimate contributes substantially to the meta-analytic OR of 1.52. The fact that the point estimate is exactly the same as the pooled estimate is noteworthy and should be double-checked and, if correct, briefly explained rather than presented without comment. Finally, the fixed-effect meta-analysis combines only three cohorts, including a small cohort of 28 patients from a non-peer-reviewed DUTRENEO preprint. Although I² = 0 is reported, this statistic has limited power to detect meaningful heterogeneity when only three studies are available, particularly when one study is very small. Please consider providing a random-effects sensitivity analysis or, at minimum, explicitly acknowledging this limitation alongside the reported I² value so that readers can appropriately calibrate their confidence in the pooled OR of 1.52.

5. The manuscript would benefit from a more explicit discussion of its relationship to very recent and closely related publications, particularly because some of the datasets overlap. First, GSE171351, which serves as the discovery cohort for the NMF-derived ecotypes in the present manuscript, is identified in Table 1 as having already been “used by Nature 2026,” referring to Zhang et al., Nature (2026), “Non-invasive profiling of the tumour microenvironment with spatial ecotypes” (Spatial EcoTyper). That study describes nine conserved spatial ecotypes and likewise uses NMF-based analysis of spatial neighborhoods, making the methodological relationship particularly relevant. Furthermore, Table 2 includes a planned “nature_SE_score” variable intended to benchmark E2/E4 against the published SE1–SE9 labels, with SE4, SE1, and SE8 specifically mentioned. However, this comparison does not appear to be reported in the Results, Tables, or Figures. Given that the Spatial EcoTyper tool is publicly available, please either (a) perform and report this comparison, including how E2/E4 relate to and potentially add value beyond the published spatial ecotypes in the same dataset, or (b) remove the unused variable from Table 2 and explicitly explain why an independent NMF pipeline was preferred over application of the existing tool. Similarly, GSE319536 originates from Yu et al., Cancer Discovery (2026), whose reported findings emphasize a luminal–basal spatial axis and TLS states rather than a CAF/TGFβ/endothelial barrier specifically. The use of this atlas for an independent hypothesis is reasonable and appropriately cited; nevertheless, please clarify the relationship between the present E2/E4 states and the spatial state calls reported in the original study, particularly if there is any substantive overlap.

6. Table 2 lists several dataset accessions—GSE326226, GSE299573, GSE305629, GSE326225, and GSE135337—that do not appear to be discussed elsewhere in the manuscript. They are described as “expected cohorts” for spatial coordinates, tissue images, and cell-type labels, but none of these datasets is described in the Methods, Table 1, or Results. In its current form, this appears to be residual material from a broader analysis template that was not fully adapted to the present study and may confuse readers and editors. Please either incorporate these cohorts into the manuscript with a clearly stated analytical role or remove them from Table 2.

7. The use of the term “underlies” in the title and abstract appears to imply a stronger mechanistic or causal interpretation than can be supported by the current study design. The analysis is retrospective, cross-sectional, and primarily transcriptomic and computational, with no perturbational experiments, functional validation, or orthogonal pathology-based validation such as IHC or multiplex immunofluorescence. The authors appropriately acknowledge this limitation in the Limitations section; however, the phrase “underlies immune exclusion and checkpoint resistance” remains more causal than the available evidence warrants. Please consider replacing this language with a more appropriately associational formulation, such as “is associated with” or “spatially co-occurs with,” throughout the title and abstract, consistent with the appropriately cautious language used in the final paragraph of the Discussion.

8. The gene-level definitions of the major signatures are not sufficiently visible in the main manuscript. The CAF/ECM, TGFβ/EMT, endothelial remodeling, hypoxia, CD8 cytotoxicity, TLS/B-cell, and related modules are central to essentially all downstream analyses, yet their specific gene lists are not provided in the manuscript body and are instead described as “locked” and apparently provided in S1 File. Please provide, at minimum in a supplementary table, the exact gene symbols included in each module, the number of genes per module, and the coverage information described in the Methods. This information is important for evaluating the quality and specificity of the signatures and for assessing potential overlap between modules. For example, some EMT-related and CAF/ECM-related genes may commonly co-occur, which is directly relevant to determining how separable the individual components of E2 actually are.

9. Please explicitly confirm whether there is any patient-level overlap among TCGABLCA, IMvigor210, GSE176307, and GSE328930. Although substantial overlap would seem unlikely, a one-line confirmation would improve transparency and is particularly important given that all four cohorts involve bladder/urothelial cancer.

10. Table 5 is relatively dense and currently combines per-cohort model comparisons with the meta-analysis results. For improved readability and interpretation, please consider separating these into two tables: one presenting the individual cohort/model results and another presenting the meta-analysis findings. This would make the distinction between cohort-specific estimates and pooled estimates clearer to readers.

11. The manuscript would benefit from thorough professional English-language editing. Several sections contain awkward or overly complex sentence structures, imprecise wording, and grammatical or stylistic issues that make some of the scientific arguments difficult to follow. Please carefully revise the manuscript for grammar, syntax, terminology, consistency, and overall readability, preferably with comprehensive editing by a fluent scientific English speaker or a professional scientific editing service. This is particularly important because the manuscript presents a relatively complex computational framework, and clearer language would substantially improve the accessibility and interpretation of the methodological and biological conclusions.

**********

what does this mean?). If published, this will include your full peer review and any attached files.

If you choose “no”, your identity will remain anonymous but your review may still be made public.

**Do you want your identity to be public for this peer review?** For information about this choice, including consent withdrawal, please see our Privacy Policy

Reviewer #1: No

Reviewer #2: **Yes:** Dr. Mohammad Amin Bakhshan

**********

[NOTE: If reviewer comments were submitted as an attachment file, they will be attached to this email and accessible via the submission site. Please log into your account, locate the manuscript record, and check for the action link "View Attachments". If this link does not appear, there are no attachment files.]

To ensure your figures meet our technical requirements, please review our figure guidelines: https://journals.plos.org/plosone/s/figures

You may also use PLOS’s free figure tool, NAAS, to help you prepare publication quality figures: https://journals.plos.org/plosone/s/figures#loc-tools-for-figure-preparation.

NAAS will assess whether your figures meet our technical requirements by comparing each figure against our figure specifications.

# Author comment: Author response to Decision Letter 1

**DOI:** 10.1371/journal.pone.0358043.r002

23 Aug 2026

Dear Academic Editor and Reviewers,

Thank you for your careful assessment and constructive comments. We have thoroughly revised the manuscript and addressed all journal requirements and reviewer comments.

A detailed point-by-point response has been uploaded separately as “PONE-D-26-26495_Response_to_Reviewers.docx.” Major revisions include recalibrating the central claims, explicitly reporting the external non-replication findings, adding NMF and neighborhood robustness analyses, performing meta-analysis and sensitivity analyses, providing complete gene-signature definitions, revising the tables and figures, rebuilding the Supporting Information archive, and improving the manuscript’s language and readability.

Both a clean revised manuscript and a marked-up version with changes have been uploaded.

Sincerely,

Yuye Wu

On behalf of all authors

Attachment Submitted filename: PONE-D-26-26495_Response_to_Reviewers.docx

# Peer review: Decision Letter 1

**DOI:** 10.1371/journal.pone.0358043.r003

**Authors:** Alireza Shoari

26 Aug 2026

A recurrent cancer-associated fibroblast/TGF-beta/endothelial barrier direction is associated with immune exclusion and checkpoint resistance in bladder cancer: a public multi-cohort transcriptomic study

PONE-D-26-26495R1

Dear Dr. Yuye,

We’re pleased to inform you that your manuscript has been judged scientifically suitable for publication and will be formally accepted for publication once it meets all outstanding technical requirements.

Within one week, you’ll receive an e-mail detailing the required amendments. When these have been addressed, you’ll receive a formal acceptance letter and your manuscript will be scheduled for publication.

An invoice will be generated when your article is formally accepted. Please note, if your institution has a publishing partnership with PLOS and your article meets the relevant criteria, all or part of your publication costs will be covered. Please make sure your user information is up-to-date by logging into Editorial Manager at Editorial Manager® and clicking the ‘Update My Information' link at the top of the page. For questions related to billing, please contact billing support.

If your institution or institutions have a press office, please notify them about your upcoming paper to help maximize its impact. If they’ll be preparing press materials, please inform our press team as soon as possible -- no later than 48 hours after receiving the formal acceptance. Your manuscript will remain under strict press embargo until 2 pm Eastern Time on the date of publication. For more information, please contact onepress@plos.org.

Kind regards,

Alireza Shoari

Academic Editor

PLOS One

Additional Editor Comments (optional):

Reviewers' comments:

Reviewer's Responses to Questions

**Comments to the Author**

Reviewer #2: All comments have been addressed

**********

2. Is the manuscript technically sound, and do the data support the conclusions?

Reviewer #2: Yes

**********

3. Has the statistical analysis been performed appropriately and rigorously?

Reviewer #2: Yes

**********

4. Have the authors made all data underlying the findings in their manuscript fully available?

The PLOS Data policy

Reviewer #2: Yes

**********

5. Is the manuscript presented in an intelligible fashion and written in standard English?

Reviewer #2: Yes

**********

Reviewer #2: (No Response)

**********

what does this mean?). If published, this will include your full peer review and any attached files.

If you choose “no”, your identity will remain anonymous but your review may still be made public.

**Do you want your identity to be public for this peer review?** For information about this choice, including consent withdrawal, please see our Privacy Policy

Reviewer #2: **Yes:** Mohammad Amin Bakhshan

**********

# Editor report: Acceptance letter

**DOI:** 10.1371/journal.pone.0358043.r004

**Authors:** Alireza Shoari

PONE-D-26-26495R1

PLOS One

Dear Dr. Wu,

I'm pleased to inform you that your manuscript has been deemed suitable for publication in PLOS One. Congratulations! Your manuscript is now being handed over to our production team.

At this stage, our production department will prepare your paper for publication. This includes ensuring the following:

* All references, tables, and figures are properly cited

* All relevant supporting information is included in the manuscript submission,

* There are no issues that prevent the paper from being properly typeset

You will receive further instructions from the production team, including instructions on how to review your proof when it is ready. Please keep in mind that we are working through a large volume of accepted articles, so please give us a few days to review your paper and let you know the next and final steps.

Lastly, if your institution or institutions have a press office, please let them know about your upcoming paper now to help maximize its impact. If they'll be preparing press materials, please inform our press team within the next 48 hours. Your manuscript will remain under strict press embargo until 2 pm Eastern Time on the date of publication. For more information, please contact onepress@plos.org.

You will receive an invoice from PLOS for your publication fee after your manuscript has reached the completed accept phase. If you receive an email requesting payment before acceptance or for any other service, this may be a phishing scheme. Learn how to identify phishing emails and protect your accounts at https://explore.plos.org/phishing.

If we can help with anything else, please email us at customercare@plos.org.

Thank you for submitting your work to PLOS One and supporting open access.

Kind regards,

PLOS One Editorial Office Staff

on behalf of

Dr. Alireza Shoari

Academic Editor

PLOS One
